# Effective reduced-order approximation for fast and robust MCRE-based parametric identification of nonlinear history-dependent material laws

**DOI:** 10.1007/s00466-026-02754-1

**Published:** 2026-02-19

**Authors:** Mainak Bhattacharyya, Ludovic Chamoin

**Affiliations:** LMPS - Laboratoire de Mécanique Paris-Saclay, Université Paris-Saclay, CentraleSupélec, ENS Paris-Saclay, CNRS, 4 Avenue des Sciences, Gif-sur-Yvette, 91190 France

**Keywords:** Parametric identification, Modified Constitutive Relation Error, Reduced order methods, Nonlinear constitutive laws, Generalised standard materials

## Abstract

This article essentially addresses the numerical frugality of model updating procedures using reduced order modelling. Nonlinear material behaviour is tackled in the article with the focus being on elasto(visco)-plasticity and elasto(visco)-plastic-damage. A proper generalised decomposition based formulation is introduced that separates the governing equations into sundered space and time problems, thereby providing low fidelity approximations. A proper orthogonal decomposition based model reduction method is also introduced to tackle variable Hooke’s tensor for softening material behaviour. The proposed methodologies are exemplified through several numerical tests in order to assess and validate performance.

## Introduction

In the context of structural health monitoring, it is essential to detect defects early on and track their growth, ensuring the integrity of structures. Such a philosophy has applications in several industrial sectors, such as transport or energy (to name a few). This necessitates the combination of numerical simulations and physical measurements, which gave rise to dynamic data driven application systems (DDDAS) (see [1, 2]), involving methods enabling real-time, dynamic information exchange between the physical system and its corresponding numerical counterpart or virtual twin (see [3] for details on twins). DDDAS attempts not only to continuously predict the evolution of the relevant physical phenomena, but also dynamically update the computational model in real-time. Such an approach assists in system adjustment as well as system diagnosis.

The coupling between measured data and constitutive modelling is generally performed by using the data to identify the parameters of the constitutive model. The identification procedure based on inverse approach exploits the affluent measurement data, which can be obtained through either digital image correlation (DIC) (see [4]), vibration measurement e.g. by means of accelerators, or optic fibre sensing (see [5]). Out of pure machine learning strategies, the parametric identification strategy essentially involves model updating based on the adjustment of the model parameters that reduces in some manner the difference between the model prediction and the measurement data. Several physics-based inverse analysis methods for model identification from full-field measurements are detailed in [6], such as the virtual field method (VFM) in [7], reciprocity gap method (RGM) in [8], finite element model updating method (FEMU) in [9], equilibrium gap method (EGM) in [10], constitutive relation error (CRE) method in [11], or modified constitutive relation error (MCRE) method in [12].

In the case of the MCRE method, the full knowledge of the measurement data and boundary conditions is not required (see [13]). Also, MCRE is able to handle spurious data due to measurement noise quite effectively (see [12–14]). The principle of MCRE consists in dividing the relations which characterise the problem into a reliable set (mechanical equilibrium) and a less reliable set (measurement data, constitutive relation, and/or boundary conditions). The reliable set is exactly satisfied, and the less reliable set is approximately satisfied through a functional minimisation. In case of CRE, only the constitutive relation is considered to be less reliable. A variant of this, referred to as optimal control (OC) method (see [15]), uses only measurement data as the less reliable set, while the reliable set includes both mechanical equilibrium and constitutive relations. MCRE is a hybrid approach, relying on both physics-based and data-based information, providing a good compromise between purely data-based approaches which require high data quantity, and model-based approaches which require low data quantity. Nevertheless, all three approaches provide estimates of the mechanical states, by means of the computations of admissible fields. [14, 16, 17] depict the usage of MCRE in the case of nonlinear parametric identifications leads to a minimisation at the mechanical model level translating to a non-standard finite element formulation, with a global (space-time) nonlinear optimisation problem. In this article, the MCRE procedure is employed for the inverse analysis.

In the nonlinear range, the resolution of the MCRE procedure incorporates an iterative procedure similar to the LATIN method (see [18]). In this article, the intent is to propose a proper generalised decomposition (PGD) based reduced order model (ROM) in space and time to be integrated in the LATIN type methodology for the MCRE process. The advantage of PGD is numerical cost effectiveness as ROMs provide low dimensional approximations for high fidelity models (see [19]); this is thus a good option to tend to real-time inverse applications with MCRE. Also, the LATIN method is inherently conducive to PGD implementation in space and time, as it is a non-incremental but iterative procedure (see [18, 20]). [21, 22] have used parametric PGDs for model updating of linear problems. A LATIN-PGD approach was used in [23] in space and time for nonlinear model updating using the aforementioned OC method. In the current article, space-time LATIN-PGD is used to solve the MCRE problem. However, unlike [23] where PGD was based on a minimisation formulation, in the current article a Galerkin formulation is used because of its computational frugality and formulation simplicity (see [24] for details on PGD formulations).

Also, as damageable materials are also studied in this article, a proper orthogonal decomposition (POD) based ROM strategy is used to approximate the variable Hooke tensor. In case of POD, certain full-order problems are computed in order to extract relevant information which can then be used to approximate the solution more efficiently (see [25]). The usage of POD in the field of mechanics deals with the creation and truncation of POD basis and coefficients in a least square sense from the snapshots of the full-order solution (see [26]).

The article starts with the problem definition (section 2), describing the governing equations. Section 3 thereafter describes the basic concept of the MCRE method, in a classical way. Section 4 provides the PGD based formulation for the MCRE procedure, which is then extended in section 5 to damageable behaviour by coupling with a POD based method. Finally the article is concluded through Section 7.

## Problem definition

**Fig. 1:**
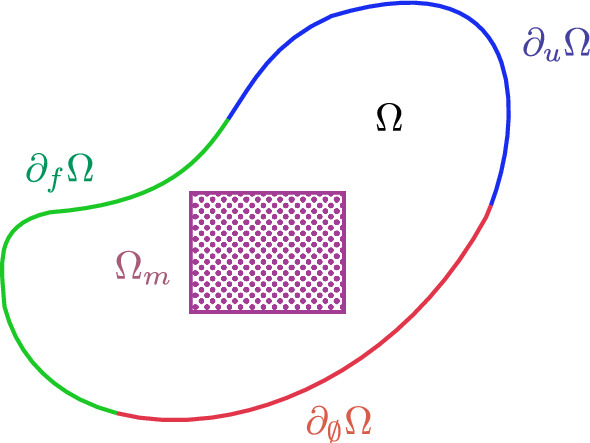
Representative schematic of the identification problem

To elucidate the basic theology of the MCRE procedure, we consider the domain $\Omega$ as shown in fig. 1. It consists of a boundary $\partial \Omega$, divided into three separate boundaries $\partial _u\Omega$, $\partial _f\Omega$, $\partial _\emptyset \Omega$, such that $\partial \Omega =\partial _u\Omega \cup \partial _f\Omega \cup \partial _\emptyset \Omega$. Dirichlet boundary conditions are applied on $\partial _u\Omega$, Neumann boundary conditions are applied on $\partial _f\Omega$, while $\partial _\emptyset \Omega$ contains no boundary information. Also, there exists a sub-domain $\Omega _m$, such that $\Omega _m\subseteq \Omega$, which represents the region where the kinematic data is measured.

For the direct mechanical problem, consider a time-dependent scenario where the time variable *t* is defined such that $t \in \left[ 0,T\right]$, with 0 and *T* the lower and upper temporal bounds. A prescribed displacement field $\underline{u}_d$ is applied on $\partial _u\Omega$, and a prescribed traction field $\underline{f}_d^s$ is applied on $\partial _f\Omega$. A body force field $\underline{f}_d^v$ can also be considered to be applied in $\Omega$.

The kinematic admissibility defines the space $\mathcal {U}_{ad}$ of displacement field $\underline{u}$ satisfying the Dirichlet boundary conditions, that is

1 $$\begin{aligned} \underline{u}\left( t\right) \vert _{\partial _u\Omega }=\underline{u}_d\left( t\right) , \, \forall t \in \left[ 0,T\right] . \end{aligned}$$

The static admissibility defines the space $\mathcal {S}_{ad}$ of stress field $\underline{\underline{\sigma }}$ (Cauchy stress) satisfying the equilibrium equation

2 $$\begin{aligned}  &   \iint _{\Omega \times \left[ 0,T\right] }\underline{\underline{\sigma }}:\underline{\underline{\varepsilon }}\left( \underline{v}\right) d\Omega dt \nonumber \\  &   \quad =\iint _{\Omega \times \left[ 0,T\right] }\underline{f}_d^v\cdot \underline{v}d\Omega dt +\iint _{\partial _f\Omega \times \left[ 0,T\right] }\underline{f}_d^s\cdot \underline{v}dS dt, \, \forall \underline{v}\in \mathcal {U}_{ad, 0}, \end{aligned}$$

where $\underline{\underline{\varepsilon }}\left( \underline{v}\right)$ is the infinitesimal strain tensor associated with the test or virtual displacement field $\underline{v}$, such that $\underline{\underline{\varepsilon }}\left( \underline{v}\right) =\nabla ^{s}\underline{v}$. The vector space $\mathcal {U}_{ad, 0}$ is the space associated with the test field vanishing on $\partial \Omega \setminus \partial _f\Omega$.

The constitutive behaviour, dictating the behaviour of the material is given by

3a $$\begin{aligned}&\underline{\underline{\sigma }}=\dfrac{\partial \psi }{\partial \underline{\underline{\varepsilon }}^e} \, \left( \text {or}\; \underline{\underline{\varepsilon }}^e=\dfrac{\partial \psi ^*}{\partial \underline{\underline{\sigma }}}\right) , \; \underline{\underline{Z}}=\dfrac{\partial \psi }{\partial \underline{\underline{X}}} \, \left( \text {or}\; \underline{\underline{X}}=\dfrac{\partial \psi ^*}{\partial \underline{\underline{Z}}} \right) \end{aligned}$$

3b $$\begin{aligned}&\underline{\underline{\sigma }}=\dfrac{\partial \phi }{\partial \dot{\underline{\underline{\varepsilon }}}^p}\, \left( \text {or}\; \dot{\underline{\underline{\varepsilon }}}^p=\dfrac{\partial \phi ^*}{\partial \underline{\underline{\sigma }}}\right) , \; \underline{\underline{Z}}{=}{-}\dfrac{\partial \phi }{\partial \dot{\underline{\underline{X}}}} \, \left( \text {or}\; \dot{\underline{\underline{X}}}{=}{-}\dfrac{\partial \phi ^*}{\partial \underline{\underline{Z}}} \right) . \end{aligned}$$

which refers to the formulation of generalized standard materials. Here, eq. (3a) refers to state equations obtained from the free energy function $\psi$, or the dual free energy function $\psi ^*$. Equation (3b) describes evolution laws obtained from the dissipation potential $\psi$, or the dual dissipation potential $\psi ^*$. The free energy function and the dissipation potential are convex scalar functions, and are related to their dual counterparts through the Legendre-Fenchel transform. The strain tensor $\underline{\underline{\varepsilon }}$ is additively decomposed into an elastic part $\underline{\underline{\varepsilon }}^e$ and an inelastic part $\underline{\underline{\varepsilon }}^p$, i.e. $\underline{\underline{\varepsilon }}=\underline{\underline{\varepsilon }}^e+\underline{\underline{\varepsilon }}^p$. The additional (internal) variables that describe the material state are represented by the set of all primal variables $\underline{\underline{X}}$ and their associated dual variables $\underline{\underline{Z}}$. The free energy function and the dissipation potential are parametrised by the set of parameters $\underline{\theta }$.

## Concept of MCRE for history-dependent materials

The MCRE aims to identify the parameters $\underline{\theta }$ of constitutive relations that fit the best to the experimental data (considered here to be the locally measured displacement field $\tilde{\underline{u}}$). This means that

4 $$\begin{aligned} \underline{u}\left( t\right) \vert _{\Omega _m}=\tilde{\underline{u}}\left( t\right) , \, \forall t \in \left[ 0,T\right] . \end{aligned}$$

Here, the Dirichlet and Neumann boundary conditions are considered to be known, however the methodology can be easily extended for unknown boundary conditions.

The general idea of the MCRE-type minimisation is to split the equations of section 2 following the reliability of information. The constitutive relation is considered as the unreliable equation and the associated constitutive relation error is defined as

5 $$\begin{aligned} \xi ^2= \int _0^T\int _\Omega \eta _\psi d\Omega dt + \int _0^T\int _0^t\int _\Omega \eta _\phi d\Omega d\tau dt, \end{aligned}$$

where the residual errors are defined as

6a $$\begin{aligned}&\eta _\psi =\psi \left( \underline{\underline{\varepsilon }}^e,\underline{\underline{X}}\right) + \psi ^*\left( \underline{\underline{\sigma }},\underline{\underline{Z}}\right) - \underline{\underline{\sigma }}:\underline{\underline{\varepsilon }}^e- \underline{\underline{Z}}\cdot \underline{\underline{X}}, \end{aligned}$$

6b $$\begin{aligned}&\eta _\phi =\phi \left( \dot{\underline{\underline{\varepsilon }}}^p,-\dot{\underline{\underline{X}}}\right) + \phi ^*\left( \underline{\underline{\sigma }},\underline{\underline{Z}}\right) - \underline{\underline{\sigma }}:\dot{\underline{\underline{\varepsilon }}}^p+ \underline{\underline{Z}}\cdot \dot{\underline{\underline{X}}}. \end{aligned}$$

The CRE concept essentially involves minimising the CRE $\xi$ over admissibility spaces to find the parameters $\underline{\theta }$ of the constitutive model. In the first idea of the method, observations were directly included in the definition of the kinematically admissible space, leading to weak robustness to measurement noise. To improve this robustness, the MCRE procedure does not impose the observations in the admissible space. This defines the MCRE functional as

7 $$\begin{aligned} \zeta ^2=\xi ^2+\dfrac{\gamma }{2}\int _0^T\int _{\Omega _m} \left\| \underline{u}-\tilde{\underline{u}} \right\| ^2 d\Omega _m dt, \end{aligned}$$

where $\gamma$ is a scaling factor.

The inverse problem thus consists of finding the optimal parameters such that

8 $$\begin{aligned} \underline{\theta }_{opt}= \min _{\underline{\theta }}\left( \min _{\varkappa \in \mathcal {A}_{ad}} \zeta ^2\left( \varkappa ,\underline{\theta }\right) \right) \end{aligned}$$

where $\mathcal {A}_{ad}$ is the admissibility space such that $\mathcal {A}_{ad} = \mathcal {U}_{ad}\times \mathcal {S}_{ad}$, while $\varkappa$ represents the whole set of state variables, i.e. $\varkappa =\left\{ \underline{\underline{\varepsilon }}^e\left( =\underline{\underline{\varepsilon }}-\underline{\underline{\varepsilon }}^p\right) ,\underline{\underline{\varepsilon }}^p,\underline{\underline{\sigma }},\underline{\underline{X}},\underline{\underline{Z}}\right\}$

The minimisation is performed sequentially and iteratively. For a given iteration *i*:

- The first step (also referred to as the basic problem) involves computing the optimal admissible state $\varkappa$ for a given parameter set $\underline{\theta }_{i-1}$, i.e.
  9 $$\begin{aligned} \varkappa _i=\min _{\varkappa \in \mathcal {A}_{ad}} \zeta ^2\left( \varkappa ,\underline{\theta }_{i-1}\right) . \end{aligned}$$
- The second step (also referred to as the identification problem) involves computing the updated material parameter by minimising the MCRE for the previously calculated admissible set, i.e.
  10 $$\begin{aligned} \underline{\theta }_i=\min _{\underline{\theta }} \zeta ^2\left( \varkappa _i,\underline{\theta }\right) . \end{aligned}$$

### The basic problem

The basic problem is the most computationally expensive part, and the resolution is performed with a strategy similar to the large time increment (LATIN) method. This is a non-incremental (i.e. global in time), and iterative method, well-suited for nonlinear problems. The method generally starts with an approximation of the quantities of interest for the whole spatial-temporal domain. Thereafter, at each iteration, the quantities of interest are improved for the whole spatial and temporal domain till a convergence is reached. In general LATIN method is based on three basic principles (see [18]).

- The first principle is the separation of difficulties. The governing equations are separated into two groups. A group of equations which are local in space, possibly non-linear; and a group of equations which are linear but possibly global in space.
- The second principle is a two-step iterative algorithm, where at each iteration the solution is constructed alternatively for the first group of equations and then for the second group of equations.
- The last principle is to solve the global problem defined on the entire space-time domain using proper generalised decomposition (PGD) based reduced order approximation such that the numerical cost is drastically lowered.

In the context of MCRE, separation of difficulties is achieved by splitting $\zeta ^2$ into two parts:

11a $$\begin{aligned}&\zeta ^2_\psi =\int _0^T\int _\Omega \eta _\psi d\Omega dt + \dfrac{\gamma }{2}\int _0^T\int _{\Omega _m} \left\| \underline{u}-\tilde{\underline{u}} \right\| ^2 d\Omega _m dt, \end{aligned}$$

11b $$\begin{aligned}&\zeta ^2_\phi =\int _0^T\int _0^t\int _\Omega \eta _\phi d\Omega d\tau dt. \end{aligned}$$

This separation enables minimising each term alternatively. The iterative minimisation is performed through a two-step methodology.

#### Global step

This step consists in the minimisation of eq. (11a), representing the state equations residual and measurement discrepancy. This minimisation is linear and global in space, under admissibility constraints. The kinematic admissibility is enforced in the discretised space as essential boundary condition. The static admissibility is enforced through a Lagrangian as

12 $$\begin{aligned} \mathcal {L}= &   \zeta ^2_\psi - \int _0^T\left[ \int _\Omega \underline{\underline{\sigma }}:\underline{\underline{\varepsilon }}\left( \underline{\lambda }\right) d\Omega - \int _\Omega \underline{f}_d^v\cdot \underline{\lambda }d\Omega \right. \nonumber \\    &   \left. - \int _{\partial _f\Omega }\underline{f}_d^s\cdot \underline{\lambda }dS \right] dt, \end{aligned}$$

where $\underline{\underline{\varepsilon }}\left( \underline{\lambda }\right)$ is the strain associated to the Lagrange multiplier field $\underline{\lambda }$.

The residual on state equations $\eta _\psi$ defined in eq. (6a) requires an inconvenient dual Legendre-Fenchel transformation (see [14]), which is necessary for the expression of $\zeta ^2_\psi$. Therefore, a displacement field $\underline{v}$ (defined up to a rigid body motion) is introduced by duality such that

13 $$\begin{aligned} \underline{\underline{\sigma }}_v=\dfrac{\partial \psi }{\partial \underline{\underline{\varepsilon }}^e_v},\; \underline{\underline{\sigma }}_v \in \mathcal {S}_{ad}. \end{aligned}$$

This therefore defines the variables associated to $\underline{v}$ as $\varkappa _v=\left\{ \underline{\underline{\varepsilon }}_v^e\left( =\underline{\underline{\varepsilon }}_v-\underline{\underline{\varepsilon }}_v^p\right) ,\underline{\underline{\varepsilon }}_v^p,\underline{\underline{\sigma }}_v,\underline{\underline{X}}_v,\underline{\underline{Z}}_v\right\}$, and variables associated to $\underline{u}$ as $\varkappa _u=\left\{ \underline{\underline{\varepsilon }}_u^e\left( =\underline{\underline{\varepsilon }}_u-\underline{\underline{\varepsilon }}_u^p\right) ,\underline{\underline{\varepsilon }}_u^p,\underline{\underline{\sigma }}_u,\underline{\underline{X}}_u,\underline{\underline{Z}}_u\right\}$. Equation (12) can be rewritten as

14 $$\begin{aligned} \mathcal {L}&=\int _0^T\int _\Omega \left[ \psi \left( \underline{\underline{\varepsilon }}_u-\underline{\underline{\varepsilon }}_u^p,\underline{\underline{X}}_u\right) -\psi \left( \underline{\underline{\varepsilon }}_v-\underline{\underline{\varepsilon }}_v^p,\underline{\underline{X}}_v\right) \right. \nonumber \\  &\quad \left. -\underline{\underline{\sigma }}_v:\left( \left( \underline{\underline{\varepsilon }}_u-\underline{\underline{\varepsilon }}_u^p\right) -\left( \underline{\underline{\varepsilon }}_v-\underline{\underline{\varepsilon }}_v^p\right) \right) -\underline{\underline{Z}}_v\cdot \left( \underline{\underline{X}}_u-\underline{\underline{X}}_v\right) \right] d\Omega dt \nonumber \\  &\quad + \dfrac{\gamma }{2}\int _0^T\int _{\Omega _m} \left\| \underline{u}-\tilde{\underline{u}} \right\| ^2 d\Omega _m dt \nonumber \\  &\quad - \int _0^T\left[ \int _\Omega \underline{\underline{\sigma }}_v:\underline{\underline{\varepsilon }}\left( \underline{\lambda }\right) d\Omega - \int _\Omega \underline{f}_d^v\cdot \underline{\lambda }d\Omega - \int _{\partial _f\Omega }\underline{f}_d^s\cdot \underline{\lambda }dS \right] dt. \end{aligned}$$

Equation (14) is a compromise between the residual on state equations and the discrepancy with measurements. Its minimisation is a linear (because elasticity is assumed to be linear here) and global in space problem. During the global step, internal variables $\underline{\underline{X}}_u$, $\underline{\underline{X}}_v$, $\underline{\underline{Z}}_u$, $\underline{\underline{Z}}_v$, $\underline{\underline{\varepsilon }}_u^p$, $\underline{\underline{\varepsilon }}_v^p$ are frozen to the value obtained at the last local step.

Stationarity of eq. (14), i.e. search of the saddle point, results into the following set of equations

15a $$\begin{aligned}&\int _0^T\int _\Omega \boldsymbol{\textrm{C}}\left( \underline{\underline{\varepsilon }}_v-\underline{\underline{\varepsilon }}_v^p \right) :\underline{\underline{\varepsilon }}\left( \delta \underline{\lambda } \right) d\Omega dt - \int _0^T\int _\Omega \underline{f}_d^v \cdot \delta \underline{\lambda } d\Omega dt \nonumber \\  &\quad - \int _0^T\int _{\partial _f\Omega }\underline{f}_d^s\cdot \delta \underline{\lambda } dS dt = 0, \end{aligned}$$

15b $$\begin{aligned}&\int _0^T\int _\Omega \boldsymbol{\textrm{C}} \left[ \left( \underline{\underline{\varepsilon }}_v-\underline{\underline{\varepsilon }}_v^p \right) -\left( \underline{\underline{\varepsilon }}_u-\underline{\underline{\varepsilon }}_u^p \right) -\underline{\underline{\varepsilon }}_{\lambda }\right] :\underline{\underline{\varepsilon }}\left( \delta \underline{v}\right) d\Omega dt=0, \end{aligned}$$

15c $$\begin{aligned}&\int _0^T\int _\Omega \boldsymbol{\textrm{C}} \left[ \left( \underline{\underline{\varepsilon }}_u-\underline{\underline{\varepsilon }}_u^p \right) - \left( \underline{\underline{\varepsilon }}_v-\underline{\underline{\varepsilon }}_v^p \right) \right] :\underline{\underline{\varepsilon }}\left( \delta \underline{u}\right) d\Omega dt \nonumber \\  &\quad + \gamma \int _0^T\int _{\Omega _m} \left( \underline{u}-\tilde{\underline{u}} \right) \cdot \delta \underline{u}d\Omega _m dt=0. \end{aligned}$$

Here, the free energy function $\psi$ is considered to be

16 $$\begin{aligned}  &   \psi \left( \underline{\underline{\varepsilon }}-\underline{\underline{\varepsilon }}^p, \underline{\underline{X}}\right) =\psi _\varepsilon \left( \underline{\underline{\varepsilon }}-\underline{\underline{\varepsilon }}^p\right) +\psi _X\left( \underline{\underline{X}}\right) , \nonumber \\    &   \quad \text {with}\; \psi _\varepsilon =\dfrac{1}{2}\left( \underline{\underline{\varepsilon }}-\underline{\underline{\varepsilon }}^p\right) :\boldsymbol{\textrm{C}}\left( \underline{\underline{\varepsilon }}-\underline{\underline{\varepsilon }}^p\right) , \end{aligned}$$

where $\boldsymbol{\textrm{C}}$ is the Hooke tensor.

Consider a particular LATIN iteration *j*; the following quantities are represented in terms of corrections, i.e.

17 $$\begin{aligned}&{\varDelta }\underline{u}_j=\underline{u}_j-\underline{u}_{j-1}, {\varDelta }\underline{v}_j=\underline{v}_j-\underline{v}_{j-1}, {\varDelta }\underline{\lambda }_j=\underline{\lambda }_j-\underline{\lambda }_{j-1}, \nonumber \\&{\varDelta }\underline{\underline{\varepsilon }}_{u\,j}=\underline{\underline{\varepsilon }}_{u\,j}-\underline{\underline{\varepsilon }}_{u\,j-1}, {\varDelta }\underline{\underline{\varepsilon }}_{v\,j}=\underline{\underline{\varepsilon }}_{v\,j}-\underline{\underline{\varepsilon }}_{v\,j-1}, \nonumber \\&{\varDelta }\underline{\underline{\varepsilon }}_{\lambda \,j}=\underline{\underline{\varepsilon }}_{\lambda \,j}-\underline{\underline{\varepsilon }}_{\lambda \,j-1}, \nonumber \\&{\varDelta }\underline{\underline{\varepsilon }}_{u\,j}^p=\underline{\underline{\varepsilon }}_{u\,j}^p-\underline{\underline{\varepsilon }}_{u\,j-1}^p, {\varDelta }\underline{\underline{\varepsilon }}_{v\,j}^p=\underline{\underline{\varepsilon }}_{v\,j}^p-\underline{\underline{\varepsilon }}_{v\,j-1}^p. \end{aligned}$$

Using these corrective terms, eq. (15b) can be rewritten as

18 $$\begin{aligned}&\int _0^T\int _\Omega \boldsymbol{\textrm{C}} \left[ \left( \underline{\underline{\varepsilon }}_{v\,j-1}-\underline{\underline{\varepsilon }}_{v\,j-1}^p \right) \right. \nonumber \\  &\quad \left. -\left( \underline{\underline{\varepsilon }}_{u\,j-1}-\underline{\underline{\varepsilon }}_{u\,j-1}^p \right) -\underline{\underline{\varepsilon }}_{\lambda \,j-1}\right] :\underline{\underline{\varepsilon }}\left( \delta \underline{v}\right) d\Omega dt \nonumber \\&\quad +\int _0^T\int _\Omega \boldsymbol{\textrm{C}} \left[ \left( {\varDelta }\underline{\underline{\varepsilon }}_{v\,j}-{\varDelta }\underline{\underline{\varepsilon }}_{v\,j}^p \right) -\left( {\varDelta }\underline{\underline{\varepsilon }}_{u\,j}-{\varDelta }\underline{\underline{\varepsilon }}_{u\,j}^p \right) \right. \nonumber \\  &\quad \left. -{\varDelta }\underline{\underline{\varepsilon }}_{\lambda \,j}\right] :\underline{\underline{\varepsilon }}\left( \delta \underline{v}\right) d\Omega dt=0. \end{aligned}$$

Now, considering at LATIN iteration $j-1$, eq. (15b) becomes

19 $$\begin{aligned}  &   \int _0^T\int _\Omega \boldsymbol{\textrm{C}} \left[ \left( \underline{\underline{\varepsilon }}_{v\,j-1}-\underline{\underline{\varepsilon }}_{v\,j-1}^p \right) \right. \nonumber \\    &   \quad \left. -\left( \underline{\underline{\varepsilon }}_{u\,j-1}-\underline{\underline{\varepsilon }}_{u\,j-1}^p \right) -\underline{\underline{\varepsilon }}_{\lambda \,j-1}\right] :\underline{\underline{\varepsilon }}\left( \delta \underline{v}\right) d\Omega dt=0. \end{aligned}$$

Using eq. (19), eq. (18) yields

20 $$\begin{aligned}  &   \left( {\varDelta }\underline{\underline{\varepsilon }}_{v\,j}-{\varDelta }\underline{\underline{\varepsilon }}_{v\,j}^p \right) -\left( {\varDelta }\underline{\underline{\varepsilon }}_{u\,j}-{\varDelta }\underline{\underline{\varepsilon }}_{u\,j}^p \right) -{\varDelta }\underline{\underline{\varepsilon }}_{\lambda \,j}=0, \end{aligned}$$

up to a rigid body motion taken here as 0. Using eq. (20), eq. (15) can be rewritten for LATIN iteration *j* as

21a $$\begin{aligned}&\int _0^T\int _\Omega \boldsymbol{\textrm{C}}\left[ \left( \underline{\underline{\varepsilon }}_{v\,j-1}-\underline{\underline{\varepsilon }}_{v\,j-1}^p \right) +\left( {\varDelta }\underline{\underline{\varepsilon }}_{u\,j}-{\varDelta }\underline{\underline{\varepsilon }}_{u\,j}^p \right) +{\varDelta }\underline{\underline{\varepsilon }}_{\lambda \,j}\right] \nonumber \\  &\quad :\underline{\underline{\varepsilon }}\left( \delta \underline{\lambda } \right) d\Omega dt \nonumber \\&\quad - \int _0^T\int _\Omega \underline{f}_d^v \cdot \delta \underline{\lambda } d\Omega dt - \int _0^T\int _{\partial _f\Omega }\underline{f}_d^s\cdot \delta \underline{\lambda } dS dt = 0, \end{aligned}$$

21b $$\begin{aligned}&\int _0^T\int _\Omega \boldsymbol{\textrm{C}} \left[ \left( \underline{\underline{\varepsilon }}_{u\,j-1}-\underline{\underline{\varepsilon }}_{u\,j-1}^p \right) - \left( \underline{\underline{\varepsilon }}_{v\,j-1}-\underline{\underline{\varepsilon }}_{v\,j-1}^p \right) - {\varDelta }\underline{\underline{\varepsilon }}_{\lambda \,j} \right] \nonumber \\  &\quad :\underline{\underline{\varepsilon }}\left( \delta \underline{u}\right) d\Omega dt \nonumber \\&\quad + \gamma \int _0^T\int _{\Omega _m} \left( \underline{u}_{j-1}+{\varDelta } \underline{u}_{j}-\tilde{\underline{u}} \right) \cdot \delta \underline{u}d\Omega _m dt=0. \end{aligned}$$

We now introduce a discretisation through finite elements such that

22 $$\begin{aligned} \underline{u}  &   =\mathbb {N}\textsf{u}, \upvarepsilon =\mathbb {B}\textsf{u}, \mathbb {K}=\underset{\text {assembled}}{\left[ \int _\Omega \mathbb {B}^T \boldsymbol{\textrm{C}}\mathbb {B} d\Omega \right] }, \mathbb {L}\nonumber \\    &   =\underset{\text {assembled}}{\left[ \int _\Omega \mathbb {B}^T \boldsymbol{\textrm{C}} d\Omega \right] }, \end{aligned}$$

where $\mathbb {N}$ is the shape function matrix, $\mathbb {B}$ involves derivatives of the shape functions, $\mathbb {K}$ is the stiffness matrix, and $\mathbb {L}$ is a similar matrix operator that projects through integration the strains at Gauss points to the nodes. The time discretisation is such that $\left\{ t_0=0,\ldots ,t_f=T\right\}$. The discretised system of equations for a particular time step $t_l$ becomes

23 $$\begin{aligned} \begin{bmatrix} \mathbb {K}_{aa} &  \mathbb {K}_{aa}\\ \left( \gamma \Pi ^T\Pi \right) _{aa} &  - \mathbb {K}_{aa} \end{bmatrix} \begin{bmatrix} {\varDelta }\textsf{u}_{a,l,j}\\ {\varDelta }\uplambda _{a,l,j} \end{bmatrix} = \begin{bmatrix} \textsf{P}\\ \textsf{Q} \end{bmatrix}, \end{aligned}$$

with

24a $$\begin{aligned}&\textsf{P}=\mathbb {L}_{ag}{\varDelta }\upvarepsilon _{u\,l,j}^p+\textsf{F}_{a,l}-\mathbb {L}_{ag}\left( \upvarepsilon _{v\,l,j-1}-\upvarepsilon _{v\,l,j-1}^p\right) \end{aligned}$$

24b $$\begin{aligned}&\textsf{Q}=\gamma \left( \left( \Pi ^T\right) _{ao}\tilde{\textsf{u}}_l- \left( \Pi ^T\Pi \right) _{ao} \textsf{u}_{l,j-1}\right) \nonumber \\&\quad - \mathbb {L}_{ag}\left( \upvarepsilon _{u\,l,j-1}-\upvarepsilon _{u\,l,j-1}^p-\upvarepsilon _{v\,l,j-1}+\upvarepsilon _{v\,l,j-1}^p\right) . \end{aligned}$$

$\textsf{u}$ is the discretised form of $\underline{u}$, i.e the vector of nodal unknowns. $\upvarepsilon$ is the discretised form of $\underline{\underline{\varepsilon }}$ (similarly, $\upvarepsilon ^p$ is that of $\underline{\underline{\varepsilon }}^p$), described at the Gauss points. $\textsf{F}$ corresponds to the nodal forces, and $\uplambda$ is the discretised version of $\underline{\lambda }$, described at the nodes.

For the initialisation of the LATIN iterations, i.e. $j=0$, it is usual to consider an elastic problem. The discretised set of equations for the elastic initialisation reads

25 $$\begin{aligned} \begin{bmatrix} \mathbb {K}_{aa} &  \mathbb {K}_{aa}\\ \left( \gamma \Pi ^T\Pi \right) _{aa} &  - \mathbb {K}_{aa} \end{bmatrix} \begin{bmatrix} \textsf{u}_{a,0}\\ \uplambda _{a,0} \end{bmatrix} = \begin{bmatrix} \textsf{F}_a-\mathbb {K}_{ad}\textsf{U}_d \\ \gamma \left( \left( \Pi ^T\right) _{ao}\tilde{\textsf{u}}- \left( \Pi ^T\Pi \right) _{ad} \textsf{U}_d\right) \end{bmatrix} . \end{aligned}$$

Here the subscript *d* denotes the nodes where the displacement is prescribed as $\textsf{U}_d$, such that $\textsf{u}_j=\left\{ \textsf{U}_d, \textsf{u}_{a,j} \right\} ^T$, where the subscript *a* represents the unprescribed nodes. For the Lagrange multiplier field, the delineation is $\uplambda _j=\left\{ 0, \uplambda _{a,j} \right\} ^T$. The corrective terms are demarcated as ${\varDelta }\textsf{u}_j=\left\{ 0, {\varDelta }\textsf{u}_{a,j} \right\} ^T$ and ${\varDelta }\uplambda _j=\left\{ 0, {\varDelta }\uplambda _{a,j} \right\} ^T$. Similarly, the force vector is delimited as $\textsf{F}=\left\{ \textsf{F}_d, \textsf{F}_{a} \right\} ^T$, where $\textsf{F}_{a}$ can contain both free edge and applied force. The operator $\Pi$ essentially projects the nodal unknowns to the measurement grid (see [12] for details), and the subscript *g* represents all the Gauss points.

Once $\textsf{u}_{0}$ and $\uplambda _{0}$ are obtained, $\textsf{v}_{0}$ is obtained as

26 $$\begin{aligned} \textsf{v}_{0}=\textsf{u}_{0}+\uplambda _{0}, \end{aligned}$$

where $\textsf{v}$ is the discretised form of $\underline{v}$.

Similarly, the resolution of ${\varDelta }\textsf{u}_{j}$, ${\varDelta }\uplambda _{j}$ from eq. (23) results in the computation of ${\varDelta }\upvarepsilon _{u\,j}$ and ${\varDelta }\upvarepsilon _{\lambda \,j}$, and thereby ${\varDelta }\upvarepsilon _{v\,j}$ is calculated through eq. (20). ${\varDelta }\textsf{v}_{a,j}$ is thereby computed as

27 $$\begin{aligned} \left[ \mathbb {K}_{aa}\right] \left\{ {\varDelta }\textsf{v}_{a,j}\right\}= &   \left\{ \mathbb {K}_{aa} {\varDelta }\textsf{u}_{a,j}+\mathbb {K}_{aa}{\varDelta }\uplambda _{a,j} \right. \nonumber \\  &   \left. +\mathbb {L}_{ag}\left( {\varDelta }\upvarepsilon ^p_{v\,j}-{\varDelta }\upvarepsilon ^p_{u\,j}\right) \right\} , \end{aligned}$$

defining ${\varDelta }\textsf{v}_j=\left\{ 0, {\varDelta }\textsf{v}_{a,j} \right\} ^T$. $\textsf{u}_j$, $\textsf{v}_j$, $\uplambda _{j}$, $\upvarepsilon _{u\,j}$, $\upvarepsilon _{v\,j}$, $\upvarepsilon _{\lambda \,j}$ are thereby calculated from eq. (17).

#### Local step

In this step, the minimisation of $\zeta ^2_\phi$ (eq. (11b)) corresponds to the integration of evolution laws at each Gauss point. The integration of the evolution laws is performed with an Euler scheme with the total strains $\underline{\underline{\varepsilon }}_u$ and $\underline{\underline{\varepsilon }}_v$ frozen to the values obtained from the last global step. The initial conditions on internal variables are enforced for the first time step. In the rate-independent case, the dissipation potential is the indicator of a convex domain and is not differentiable, whereas in the rate-dependent case, this potential is differentiable.

The solution strategy is similar to an elastic predictor-(visco)plastic corrector type methodology, with the total strains ($\upvarepsilon _{u}$, $\upvarepsilon _{v}$) obtained from the last global stage. The initial condition has to be imposed in this stage.

Consider, for instance, a particular time step $t_l$ and a particular LATIN iteration *j*. For any particular Gauss point, with $w=\left\{ u,v \right\}$, the elastic prediction involves initialising the variables as

28a $$\begin{aligned}&\upvarepsilon ^p_{w\,l,j}=\upvarepsilon ^p_{w\,l-1,j}, \textsf{X}_{w\,l,j}=\textsf{X}_{w\,l-1,j}, \end{aligned}$$

28b $$\begin{aligned}&\textsf{Z}_{w\,l,j}=\dfrac{\partial \psi _X}{\partial \underline{\underline{X}}}\vert _{\textsf{X}_{w\,l,j}}, \upsigma _{w\,l,j}=\dfrac{\partial \psi _\varepsilon }{\partial \left( \underline{\underline{\varepsilon }}-\underline{\underline{\varepsilon }}^p\right) }\vert _{\upvarepsilon _{w\,l,j-1}, \upvarepsilon ^p_{w\,l,j}}. \end{aligned}$$

The next step is to check the yield criterion. If the yield function $f_{w,y} \ge 0$, then the variables are updated by solving the following set of equations,

29a $$\begin{aligned} \upsigma _{w\,l,j}&=\dfrac{\partial \psi _\varepsilon }{\partial \left( \underline{\underline{\varepsilon }}-\underline{\underline{\varepsilon }}^p\right) }\vert _{\upvarepsilon _{w\,l,j-1}, \upvarepsilon ^p_{w\,l,j}}, \textsf{Z}_{w\,l,j}=\dfrac{\partial \psi _X}{\partial \underline{\underline{X}}}\vert _{\textsf{X}_{w\,l,j}}, \end{aligned}$$

29b $$\begin{aligned} \upvarepsilon ^p_{w\,l,j}&=\upvarepsilon ^p_{w\,l-1,j} +{\varDelta } t_l \dfrac{\partial \phi ^*}{\partial \underline{\underline{\sigma }}}\vert _{\upsigma _{w\,l,j}, \textsf{Z}_{w\,l,j}}, \textsf{X}_{w\,l,j}\nonumber \\  &=\textsf{X}_{w\,l-1,j} -{\varDelta } t_l \dfrac{\partial \phi ^*}{\partial \underline{\underline{Z}}}\vert _{\upsigma _{w\,l,j}, \textsf{Z}_{w\,l,j}}, \end{aligned}$$

with the time step size ${\varDelta } t_l=t_l-t_{l-1}$. Here the Euler implicit method is used to integrate the first order ODEs, however other methods can also be used. The nonlinear equations (eq. (29)) are to be solved simultaneously using any nonlinear solver. Here $\textsf{X}, \textsf{Z}, \upsigma$ are the discretised versions of $\underline{\underline{X}}, \underline{\underline{Z}}, \underline{\underline{\sigma }}$, defined at the Gauss points.

It has to be mentioned that for the initialisation step ($j=0$), the internal variables $\upvarepsilon ^p_{u\,0},\upvarepsilon ^p_{v\,0},\textsf{X}_{u\,0},\textsf{X}_{v\,0},\textsf{Z}_{u\,0},\textsf{Z}_{v\,0}$ are considered to be 0, and the stress variables are obtained as

30 $$\begin{aligned} \upsigma _{u\,0}=\dfrac{\partial \psi _\varepsilon }{\partial \left( \underline{\underline{\varepsilon }}-\underline{\underline{\varepsilon }}^p\right) }\vert _{\upvarepsilon _{u\,0}, \upvarepsilon ^p_{u\,0}}, \upsigma _{v\,0}=\dfrac{\partial \psi _\varepsilon }{\partial \left( \underline{\underline{\varepsilon }}-\underline{\underline{\varepsilon }}^p\right) }\vert _{\upvarepsilon _{v\,0}, \upvarepsilon ^p_{v\,0}}. \end{aligned}$$

The stopping criterion (for the iterative process) is essentially defined through a LATIN error indicator $\ell$ obtained as

31 $$\begin{aligned} \ell&=\left[ \dfrac{\int _0^T\int _\Omega \left( \left( \underline{\underline{\varepsilon }}_{v\,j}-\underline{\underline{\varepsilon }}_{v\,j-1}\right) :\left( \underline{\underline{\varepsilon }}_{v\,j}-\underline{\underline{\varepsilon }}_{v\,j-1}\right) \right) d\Omega dt}{\dfrac{1}{2}\int _0^T\int _\Omega \left( \underline{\underline{\varepsilon }}_{v\,j}:\underline{\underline{\varepsilon }}_{v\,j}+\underline{\underline{\varepsilon }}_{v\,j-1}:\underline{\underline{\varepsilon }}_{v\,j-1}\right) d\Omega dt}\right. \nonumber \\&\quad + \left. \dfrac{\int _0^T\int _\Omega \left( \left( \underline{\underline{\varepsilon }}_{u\,j}-\underline{\underline{\varepsilon }}_{u\,j-1}\right) :\left( \underline{\underline{\varepsilon }}_{u\,j}-\underline{\underline{\varepsilon }}_{u\,j-1}\right) \right) d\Omega dt}{\dfrac{1}{2}\int _0^T\int _\Omega \left( \underline{\underline{\varepsilon }}_{u\,j}:\underline{\underline{\varepsilon }}_{u\,j}+\underline{\underline{\varepsilon }}_{u\,j-1}:\underline{\underline{\varepsilon }}_{u\,j-1}\right) d\Omega dt}\right] ^{1/2}, \end{aligned}$$

and if this is less than a certain pre-determined value, the LATIN iteration is considered to be converged.

### The identification problem

In the proposed approach, the identification problem of the inverse problem is the minimisation of the MCRE functional ($\zeta ^2$) with respect to parameters. In order to solve this minimisation problem, the various optimisation methods available in the literature can be used. A convenient approach is to use the optimal admissible set (as given in section 3.1), thereby it is natural to solve the minimisation problem eq. (10) using the gradient descent approach. The update of the parameters is given as

32 $$\begin{aligned} \underline{\theta }_i=\underline{\theta }_{i-1}-\hslash \dfrac{d \zeta ^2\left( \varkappa _i,\underline{\theta } \right) }{d\underline{\theta }}. \end{aligned}$$

Here, $\hslash$ is the step-size of the gradient descent. The gradient is computed through the adjoint-state approach and eq. (32) can be rewritten as

33 $$\begin{aligned} \underline{\theta }_i=\underline{\theta }_{i-1}-\hslash \dfrac{\partial \zeta ^2\left( \varkappa _i,\underline{\theta } \right) }{\partial \underline{\theta }}. \end{aligned}$$

The succinct procedure of the total inverse problem is given in algorithm 1.

**Algorithm 1:**
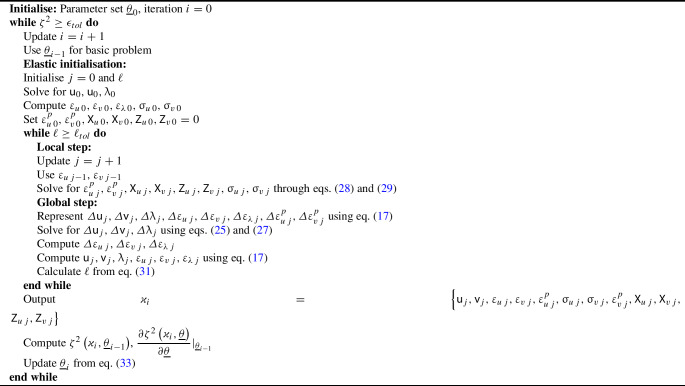
MCRE method for inverse analysis

## PGD for the basic problem

The main purpose of reduced order modelling (ROM) is to provide a frugal resolution technique. The idea of model reduction is to seek the solution in a reduced-order basis, whose dimension is much smaller than the size of the original high-dimensional model. As mentioned before, the basic problem is the most expensive part of the inverse problem, thereby the idea herein is to propose a reduced order methodology that reduces its cost.

Proper generalised decomposition (PGD) consists in seeking the solution of a problem in a relevant reduced-order basis but this one is generated automatically and on-the-fly by a greedy algorithm, simultaneously with the successive approximations of the solution. PGD is a quite obvious choice among reduced order approaches as it is extremely conducive to LATIN-type approach and does not require any separate training phase for generating the reduced order basis.

In the context of the inverse problem described in section 3, the idea is to separate the space-time problem into segregated space and time problems. This is essentially done by representing the quantities of interest into separated variable forms (as functions of space and time).

Consider first the global stage of the basic problem, especially the quantities of interest ${\varDelta }\underline{\lambda }_j$ and ${\varDelta }\underline{u}_j$, for a particular LATIN iteration *j*. Usually there are two options, either add new reduced order bases (i.e add new space-time pairs), or update the existing reduced order bases (i.e update only the time functions).

### Addition of space-time modes

Considering that the variables $\underline{\lambda }_{j-1}$ and $\underline{u}_{j-1}$ are approximated as

34 $$\begin{aligned} \underline{\lambda }_{j-1}  &   =\underline{\lambda }_{0}+\sum _{\imath =1}^{\varpi -1}\underline{\overline{{\varLambda }}}^\imath \left( \Omega \right) \alpha ^\imath _\lambda \left( t\right) , \nonumber \\    &   \quad \underline{u}_{j-1}=\underline{u}_{0}+\sum _{\jmath =1}^{\varsigma -1}\underline{\overline{U}}^\jmath \left( \Omega \right) \alpha ^\jmath _u\left( t\right) , \end{aligned}$$

the variables ${\varDelta }\underline{\lambda }_j$ and ${\varDelta }\underline{u}_j$ are represented as

35 $$\begin{aligned} {\varDelta }\underline{\lambda }_j\left( \Omega ,t\right) =\underline{\overline{{\varLambda }}}^\varpi \left( \Omega \right) \alpha _\lambda ^\varpi \left( t\right) ,{\varDelta } \underline{u}\left( \Omega ,t\right) =\underline{\overline{U}}^\varsigma \left( \Omega \right) \alpha _u^\varsigma \left( t\right) , \end{aligned}$$

where $\varpi$ and $\varsigma$ are the numbers of modes. $(\underline{\overline{{\varLambda }}}, \underline{\overline{U}})$ represent space functions, while $(\alpha _\lambda , \alpha _u)$ represent time functions. It has to be mentioned that according to eq. (35), only one new space-time is being added per quantity of interest for a given LATIN iteration *j* (i.e. $\varpi$th mode for ${\varDelta }\underline{\lambda }_j$ and $\varsigma$th mode for ${\varDelta }\underline{u}_j$). The subscript *j* is dropped for ${\varDelta }\underline{\lambda }_j$ and ${\varDelta }\underline{u}_j$, and the subscripts $\varpi ,\varsigma$ are dropped for $\underline{\overline{{\varLambda }}}, \underline{\overline{U}}$, $\alpha _u$, and $\alpha _\lambda$ for the subsequent developments, for the sake of clarity.

Considering now eq. (21), $\delta \underline{\lambda }$ and $\delta \underline{u}$ are represented through separated variable form as

36 $$\begin{aligned} \delta \underline{\lambda }= \underline{\overline{{\varLambda }}} \delta \alpha _\lambda + \alpha _\lambda \delta \underline{\overline{{\varLambda }}}, \delta \underline{u}= \underline{\overline{U}} \delta \alpha _u + \alpha _u \delta \underline{\overline{U}}. \end{aligned}$$

Also, the corresponding strains are written as

37 $$\begin{aligned} {\varDelta }\underline{\underline{\varepsilon }}_\lambda \left( \Omega ,t\right) =\overline{\underline{\underline{\varepsilon }}}_\lambda \left( \Omega \right) \alpha _\lambda \left( t\right) ,{\varDelta }\underline{\underline{\varepsilon }}_u\left( \Omega ,t\right) =\overline{\underline{\underline{\varepsilon }}}_u\left( \Omega \right) \alpha _u\left( t\right) , \end{aligned}$$

where $\overline{\underline{\underline{\varepsilon }}}_\lambda ,\overline{\underline{\underline{\varepsilon }}}_u$ are space functions.

Using eq. (36), eq. (21) can be re-written as a space problem of the form

38a $$\begin{aligned}&\int _\Omega \boldsymbol{\textrm{C}}\left[ \overline{\underline{\underline{\varepsilon }}}_u \beta +\overline{\underline{\underline{\varepsilon }}}_\lambda \kappa \right] :\underline{\underline{\varepsilon }}\left( \delta \underline{\overline{{\varLambda }}} \right) d\Omega \nonumber \\&\quad +\int _\Omega \boldsymbol{\textrm{C}}\int _0^T\left( \left( \underline{\underline{\varepsilon }}_{v\,j-1}-\underline{\underline{\varepsilon }}_{v\,j-1}^p -{\varDelta }\underline{\underline{\varepsilon }}_{u\,j}^p \right) \alpha _\lambda \right) dt :\underline{\underline{\varepsilon }}\left( \delta \underline{\overline{{\varLambda }}} \right) d\Omega \nonumber \\&\quad - \int _\Omega \int _0^T\left( \underline{f}_d^v \alpha _\lambda \right) dt \cdot \delta \underline{\overline{{\varLambda }}} d\Omega \nonumber \\  &\quad - \int _{\partial _f\Omega }\int _0^T\left( \underline{f}_d^s\alpha _\lambda \right) dt\cdot \delta \underline{\overline{{\varLambda }}} dS = 0, \end{aligned}$$

38b $$\begin{aligned}&\int _{\Omega _m} \left( \underline{\overline{U}}\eta \right) \cdot \delta \underline{\overline{U}} d\Omega _m - \int _\Omega \boldsymbol{\textrm{C}} \left( \overline{\underline{\underline{\varepsilon }}}_\lambda \beta \right) : \underline{\underline{\varepsilon }}\left( \delta \underline{\overline{U}}\right) d\Omega \nonumber \\&\quad + \int _\Omega \boldsymbol{\textrm{C}} \int _0^T\left( \left( \underline{\underline{\varepsilon }}_{u\,j-1}-\underline{\underline{\varepsilon }}_{u\,j-1}^p \right. \right. \nonumber \\&\quad - \left. \left. \underline{\underline{\varepsilon }}_{v\,j-1}+\underline{\underline{\varepsilon }}_{v\,j-1}^p \right) \alpha _u\right) dt: \underline{\underline{\varepsilon }}\left( \delta \underline{\overline{U}}\right) d\Omega \nonumber \\  &\quad + \gamma \int _{\Omega _m} \int _0^T\left( \left( \underline{u}_{j-1}-\tilde{\underline{u}} \right) \alpha _u\right) dt \cdot \delta \underline{\overline{U}} d\Omega _m =0, \end{aligned}$$

with

39 $$\begin{aligned} \beta= &   \int _0^T\left( \alpha _u \alpha _\lambda \right) dt, \kappa =\int _0^T\left( \alpha _\lambda \alpha _\lambda \right) dt, \nonumber \\ \eta= &   \gamma \int _0^T\left( \alpha _u \alpha _u \right) dt, \end{aligned}$$

and a time problem,

40a $$\begin{aligned}&\int _0^T\left[ \alpha _u \Upsilon +\alpha _\lambda \Gamma \right] \delta \alpha _\lambda dt \nonumber \\&\quad +\int _0^T\int _\Omega \left( \boldsymbol{\textrm{C}}\left( \underline{\underline{\varepsilon }}_{v\,j-1}-\underline{\underline{\varepsilon }}_{v\,j-1}^p -{\varDelta }\underline{\underline{\varepsilon }}_{u\,j}^p \right) :\overline{\underline{\underline{\varepsilon }}}_\lambda \right) d\Omega \delta \alpha _\lambda dt \nonumber \\&\quad - \int _0^T \int _\Omega \left( \underline{f}_d^v\cdot \underline{\overline{{\varLambda }}} \right) d\Omega \delta \alpha _\lambda dt \nonumber \\  &\quad - \int _0^T \int _{\partial _f\Omega }\left( \underline{f}_d^s\cdot \underline{\overline{{\varLambda }}} \right) dS \delta \alpha _\lambda dt = 0, \end{aligned}$$

40b $$\begin{aligned}&\int _0^T \left( \alpha _u \Psi \right) \delta \alpha _u dt - \int _0^T \left( \alpha _\lambda \Phi \right) \delta \alpha _u dt \nonumber \\  &\quad + \int _0^T \int _\Omega \left( \boldsymbol{\textrm{C}}\left( \underline{\underline{\varepsilon }}_{u\,j-1}-\underline{\underline{\varepsilon }}_{u\,j-1}^p \right. \right. \nonumber \\&\quad - \left. \left. \underline{\underline{\varepsilon }}_{v\,j-1}+\underline{\underline{\varepsilon }}_{v\,j-1}^p \right) : \overline{\underline{\underline{\varepsilon }}}_u\right) d\Omega \delta \alpha _u dt \nonumber \\  &\quad + \gamma \int _0^T \int _{\Omega _m} \left( \left( \underline{u}_{j-1}-\tilde{\underline{u}} \right) \cdot \underline{\overline{U}} \right) d\Omega _m \delta \alpha _u dt =0 , \end{aligned}$$

with

41 $$\begin{aligned}&\Upsilon =\int _\Omega \left( \boldsymbol{\textrm{C}}\overline{\underline{\underline{\varepsilon }}}_u : \overline{\underline{\underline{\varepsilon }}}_\lambda \right) d\Omega , \Gamma =\int _\Omega \left( \boldsymbol{\textrm{C}}\overline{\underline{\underline{\varepsilon }}}_\lambda : \overline{\underline{\underline{\varepsilon }}}_\lambda \right) d\Omega , \nonumber \\&\Psi =\gamma \int _{\Omega _m}\left( \underline{\overline{U}} \cdot \underline{\overline{U}} \right) d\Omega _m, \Phi =\int _\Omega \left( \boldsymbol{\textrm{C}}\overline{\underline{\underline{\varepsilon }}}_\lambda : \overline{\underline{\underline{\varepsilon }}}_u \right) d\Omega . \end{aligned}$$

Using the finite element discretisation, the space problem reads

42 $$\begin{aligned} \begin{bmatrix} \beta \mathbb {K}_{aa} &  \kappa \mathbb {K}_{aa}\\ \eta \left( \Pi ^T\Pi \right) _{aa} &  - \beta \mathbb {K}_{aa} \end{bmatrix} \begin{bmatrix} \overline{\textsf{U}}_{a}\\ \overline{\Lambda }_{a} \end{bmatrix} = \begin{bmatrix} \textsf{M}_a\\ \textsf{N}_a \end{bmatrix}, \end{aligned}$$

with

43a $$\begin{aligned}&\textsf{M}=\lfloor \left( \mathbb {L}{\varDelta }\upvarepsilon _{u\,l,j}^p+\textsf{F}_l-\mathbb {L}\left( \upvarepsilon _{v\,l,j-1}-\upvarepsilon _{v\,l,j-1}^p \right) \right) \upalpha _{\lambda \,l} \rfloor , \end{aligned}$$

43b $$\begin{aligned}&\textsf{N}=\lfloor \left( \gamma \left( \Pi ^T\tilde{\textsf{u}}_l- \Pi ^T\Pi \textsf{u}_{l,j-1}\right) \right. \nonumber \\&\quad \left. - \mathbb {L}\left( \upvarepsilon _{u\,l,j-1}-\upvarepsilon _{u\,l,j-1}^p-\upvarepsilon _{v\,l,j-1}+\upvarepsilon _{v\,l,j-1}^p\right) \right) \upalpha _{u\,l} \rfloor , \end{aligned}$$

where $l=\left\{ 0, \ldots , f \right\}$, and $\lfloor \blacksquare \rfloor$ represents integrations with respect to time. Equation (39) can also be written in discretised form, similar to eq. (43). These integrations can be solved by any numerical integration method (for instance the trapezoidal rule). Also, the subscript *a* follows the same definition introduced in section 3.1. $\overline{\textsf{U}}, \overline{\Lambda }$ are the discretised forms of $\underline{\overline{U}}, \underline{\overline{{\varLambda }}}$ at the nodes, and $\upalpha _u, \upalpha _\lambda$ are the discretised forms of $\alpha _u, \alpha _\lambda$ at the time steps.

The time problem can similarly be written for a particular time step $t_l$ as

44 $$\begin{aligned} \begin{bmatrix} \Upsilon &  \Gamma \\ \Psi &  - \Phi \end{bmatrix} \begin{bmatrix} \upalpha _{u \,l}\\ \upalpha _{\lambda \,l} \end{bmatrix} = \begin{bmatrix} \textsf{G}_l\\ \textsf{H}_l \end{bmatrix}, \end{aligned}$$

with

45 $$\begin{aligned} \Upsilon =\overline{\Lambda }^T\mathbb {K}\overline{\textsf{U}}, \Gamma =\overline{\Lambda }^T\mathbb {K}\overline{\Lambda }, \Psi = \gamma \overline{\textsf{U}}^T \Pi ^T\Pi \overline{\textsf{U}}, \Phi = \overline{\textsf{U}}^T\mathbb {K}\overline{\Lambda }, \end{aligned}$$

and

46a $$\begin{aligned}&\textsf{G}_l=\overline{\Lambda }^T\left( \mathbb {L}{\varDelta }\upvarepsilon _{u\,l,j}^p+\textsf{F}_l-\mathbb {L}\left( \upvarepsilon _{v\,l,j-1}-\upvarepsilon _{v\,l,j-1}^p \right) \right) , \end{aligned}$$

46b $$\begin{aligned}&\textsf{H}_l= \overline{\textsf{U}}^T\left( \gamma \left( \Pi ^T\tilde{\textsf{u}}_l- \Pi ^T\Pi \textsf{u}_{l,j-1}\right) \right. \nonumber \\  &\quad \left. - \mathbb {L}\left( \upvarepsilon _{u\,l,j-1}-\upvarepsilon _{u\,l,j-1}^p-\upvarepsilon _{v\,l,j-1}+\upvarepsilon _{v\,l,j-1}^p\right) \right) . \end{aligned}$$

The space problem (eq. (42)) and the time problem (eq. (44)) are solved through a fixed point iteration. The iteration is initialised by arbitrary time functions and thereafter eqs. (42) and (44) are solved iteratively till convergence is achieved.

The new space functions are orthonormalised with respect to the previously existing spatial bases through the Gram-Schmidt method, in order to prevent over expansion of the number of basis functions with low contribution for some of them, that may lead to a useless increase of the computation cost. Also, in the numerical process all former time functions are updated. The new time functions are thus modified and the newly added space-time pair may be rejected if the corresponding modified time function has an insignificant norm. Interested readers may consult [27] for details on this process.

### Update of time functions

Other than expanding the reduced order bases, the second option is to update the existing bases. In that case, considering the variables $\underline{\lambda }_{j-1}$ and $\underline{u}_{j-1}$ described according to eq. (34), the corrective terms defined in eq. (35) are approximated as

47 $$\begin{aligned} {\varDelta }\underline{\lambda }_{j-1}= &   \sum _{\imath =1}^{\varpi -1}\underline{\overline{{\varLambda }}}^\imath \left( \Omega \right) {\varDelta }\alpha ^\imath _\lambda \left( t\right) , \nonumber \\ {\varDelta }\underline{u}_{j-1}= &   \sum _{\jmath =1}^{\varsigma -1}\underline{\overline{U}}^\jmath \left( \Omega \right) {\varDelta }\alpha ^\jmath _u\left( t\right) . \end{aligned}$$

Here, $\left\{ {\varDelta }\alpha _\lambda ^\imath \right\} _{\imath =1}^{\varpi -1}, \left\{ {\varDelta }\alpha _u^\jmath \right\} _{\jmath =1}^{\varsigma -1}$ are corrections to the time functions, such that

48a $$\begin{aligned}&\alpha _{\lambda \,\text {up}}^{\imath }=\alpha _\lambda ^{\imath }+{\varDelta }\alpha _\lambda ^{\imath }, \text {with}\; \imath =\lbrace 1,\ldots ,\varpi -1\rbrace , \end{aligned}$$

48b $$\begin{aligned}&\alpha _{u\,\text {up}}^{\jmath }=\alpha _u^{\jmath }+{\varDelta }\alpha _u^{\jmath }, \text {with}\; \jmath =\lbrace 1,\ldots ,\varsigma -1\rbrace . \end{aligned}$$

The updated functions are represented by $\left\{ \alpha _{\lambda \,\text {up}}^\imath \right\} _{\imath =1}^{\varpi -1}, \left\{ \alpha _{u\,\text {up}}^\jmath \right\} _{\jmath =1}^{\varsigma -1}$, and the total quantities of interest become

49 $$\begin{aligned} \underline{\lambda }_{j}=\underline{\lambda }_{0}+\sum _{\imath =1}^{\varpi -1}\underline{\overline{{\varLambda }}}^\imath \left( \Omega \right) \alpha ^\imath _\lambda \left( t\right) , \underline{u}_{j}=\underline{u}_{0}+\sum _{\jmath =1}^{\varsigma -1}\underline{\overline{U}}^\jmath \left( \Omega \right) \alpha ^\jmath _u\left( t\right) , \end{aligned}$$

where the time functions are updated functions as per eq. (48).

The problem to be solved is the time problem with known space functions with the quantities of interest being $\left\{ {\varDelta }\alpha _\lambda ^\imath \right\} _{\imath =1}^{\varpi -1}, \left\{ {\varDelta }\alpha _u^\jmath \right\} _{\jmath =1}^{\varsigma -1}$. The time problem of eq. (40) can be rewritten as

50a $$\begin{aligned}&\int _0^T \begin{bmatrix} \delta {\varDelta }\alpha _\lambda ^{1}&\dots&\delta {\varDelta }\alpha _\lambda ^{\varpi -1} \end{bmatrix}\nonumber \\  &\left( \begin{bmatrix} \Upsilon _{1,1} &  \dots &  \Upsilon _{1,\varsigma -1}\\ \vdots &  \ddots &  \vdots \\ \Upsilon _{\varpi -1,1} &  \dots &  \Upsilon _{\varpi -1,\varsigma -1} \end{bmatrix} \begin{bmatrix} {\varDelta }\alpha _u^{1}\\ \vdots \\ {\varDelta }\alpha _u^{\varsigma -1} \end{bmatrix} \right. \nonumber \\&\quad +\begin{bmatrix} \Gamma _{1,1} &  \dots &  \Gamma _{1,\varpi -1}\\ \vdots &  \ddots &  \vdots \\ \Gamma _{\varpi -1,1} &  \dots &  \Gamma _{\varpi -1,\varpi -1} \end{bmatrix} \begin{bmatrix} {\varDelta }\alpha _\lambda ^{1}\\ \vdots \\ {\varDelta }\alpha _\lambda ^{\varpi -1} \end{bmatrix}\nonumber \\&\quad +\int _\Omega \left( \boldsymbol{\textrm{C}}\left( \underline{\underline{\varepsilon }}_{v\,j-1}-\underline{\underline{\varepsilon }}_{v\,j-1}^p -{\varDelta }\underline{\underline{\varepsilon }}_{u\,j}^p \right) : \begin{bmatrix} \overline{\underline{\underline{\varepsilon }}}_{\lambda }^1\\ \vdots \\ \overline{\underline{\underline{\varepsilon }}}_{\lambda }^{\varpi -1} \end{bmatrix} \right) d\Omega \nonumber \\&\quad - \left. \int _\Omega \left( \underline{f}_d^v\cdot \begin{bmatrix} \underline{\overline{{\varLambda }}}^1\\ \vdots \\ \underline{\overline{{\varLambda }}}^{\varpi -1} \end{bmatrix} \right) d\Omega - \int _{\partial _f\Omega }\left( \underline{f}_d^s\cdot \begin{bmatrix} \underline{\overline{{\varLambda }}}^1\\ \vdots \\ \underline{\overline{{\varLambda }}}^{\varpi -1} \end{bmatrix} \right) dS \right) dt = 0, \end{aligned}$$

50b $$\begin{aligned}&\int _0^T \begin{bmatrix} \delta {\varDelta }\alpha _u^{1}&\dots&\delta {\varDelta }\alpha _u^{\varsigma -1} \end{bmatrix} \left( \begin{bmatrix} \Psi _{1,1} &  \dots &  \Psi _{1,\varsigma -1}\\ \vdots &  \ddots &  \vdots \\ \Psi _{\varsigma -1,1} &  \dots &  \Psi _{\varsigma -1,\varsigma -1} \end{bmatrix} \begin{bmatrix} {\varDelta }\alpha _u^{1}\\ \vdots \\ {\varDelta }\alpha _u^{\varsigma -1} \end{bmatrix} \right. \nonumber \\&\quad -\begin{bmatrix} \Phi _{1,1} &  \dots &  \Phi _{1,\varpi -1}\\ \vdots &  \ddots &  \vdots \\ \Phi _{\varsigma -1,1} &  \dots &  \Phi _{\varsigma -1,\varpi -1} \end{bmatrix} \begin{bmatrix} {\varDelta }\alpha _\lambda ^{1}\\ \vdots \\ {\varDelta }\alpha _\lambda ^{\varpi -1} \end{bmatrix}\nonumber \\&\quad +\int _\Omega \left( \boldsymbol{\textrm{C}}\left( \underline{\underline{\varepsilon }}_{u\,j-1}-\underline{\underline{\varepsilon }}_{u\,j-1}^p -\underline{\underline{\varepsilon }}_{v\,j-1}+\underline{\underline{\varepsilon }}_{v\,j-1}^p \right) : \begin{bmatrix} \overline{\underline{\underline{\varepsilon }}}_{\lambda }^1\\ \vdots \\ \overline{\underline{\underline{\varepsilon }}}_{\lambda }^{\varpi -1} \end{bmatrix} \right) d\Omega \nonumber \\&\quad + \left. \gamma \int _{\Omega _m}\left( \left( \underline{u}_{j-1}-\tilde{\underline{u}} \right) \cdot \begin{bmatrix} \underline{\overline{U}}_1\\ \vdots \\ \underline{\overline{U}}_{\varsigma -1} \end{bmatrix} \right) d\Omega _m \right) dt = 0, \end{aligned}$$

with

51 $$\begin{aligned}&\Upsilon _{\imath ,\jmath }=\int _\Omega \left( \boldsymbol{\textrm{C}}\overline{\underline{\underline{\varepsilon }}}_{u}^{\jmath } : \overline{\underline{\underline{\varepsilon }}}_{\lambda }^{\imath } \right) d\Omega , \Gamma _{\imath ,\imath ^\prime }=\int _\Omega \left( \boldsymbol{\textrm{C}}\overline{\underline{\underline{\varepsilon }}}_{\lambda }^{\imath ^\prime } : \overline{\underline{\underline{\varepsilon }}}_{\lambda }^{\imath } \right) d\Omega , \nonumber \\&\Psi {\jmath ,\jmath ^\prime }=\gamma \int _{\Omega _m}\left( \underline{\overline{U}}^{\jmath ^\prime } \cdot \underline{\overline{U}}^{\jmath } \right) d\Omega _m, \nonumber \\  &\Phi _{\jmath ,\imath }=\int _\Omega \left( \boldsymbol{\textrm{C}}\overline{\underline{\underline{\varepsilon }}}_{\lambda }^{\imath } : \overline{\underline{\underline{\varepsilon }}}_{u}^{\jmath } \right) d\Omega , \end{aligned}$$

where the indices are $\imath , \imath ^\prime =\left\{ 1,\ldots , \varpi -1\right\}$ and $\jmath , \jmath ^\prime =\left\{ 1,\ldots , \varsigma -1\right\}$.

The corresponding discretised equation for a time step $t_l$ can be written as

52 $$\begin{aligned} \begin{bmatrix} \left[ \Upsilon \right] &  \left[ \Gamma \right] \\ \left[ \Psi \right] &  - \left[ \Phi \right] \end{bmatrix} \begin{bmatrix} \left\{ {\varDelta }\upalpha _u\right\} _l\\ \left\{ {\varDelta }\upalpha _\lambda \right\} _l \end{bmatrix} = \begin{bmatrix} \left\{ \textsf{G}\right\} _l\\ \left\{ \textsf{H}\right\} _l \end{bmatrix}, \end{aligned}$$

with

53 $$\begin{aligned} \Upsilon _{\imath ,\jmath }= &   \overline{\Lambda }_{\imath }^T\mathbb {K}\overline{\textsf{U}}_{\jmath }, \nonumber \\ \Gamma _{\imath ,\imath ^\prime }= &   \overline{\Lambda }_{\imath }^T\mathbb {K}\overline{\Lambda }_{\imath ^\prime }, \Psi _{\jmath ,\jmath ^\prime }= \gamma \overline{\textsf{U}}_{\jmath }^T \Pi ^T\Pi \overline{\textsf{U}}_{\jmath ^\prime }, \Phi _{\jmath ,\imath }= \overline{\textsf{U}}_{\jmath }^T\mathbb {K}\overline{\Lambda }_{\imath }, \end{aligned}$$

and

54a $$\begin{aligned}&\textsf{G}_{\imath ,l}=\overline{\Lambda }_{\imath }^T\left( \mathbb {L}{\varDelta }\upvarepsilon _{u\,l,j}^p+\textsf{F}_l-\mathbb {L}\left( \upvarepsilon _{v\,l,j-1}-\upvarepsilon _{v\,l,j-1}^p \right) \right) , \end{aligned}$$

54b $$\begin{aligned}&\textsf{H}_{\jmath ,l}= \overline{\textsf{U}}_{\jmath }^T\left( \gamma \left( \Pi ^T\tilde{\textsf{u}}_l- \Pi ^T\Pi \textsf{u}_{l,j-1}\right) \right. \nonumber \\  &\quad \left. - \mathbb {L}\left( \upvarepsilon _{u\,l,j-1}-\upvarepsilon _{u\,l,j-1}^p-\upvarepsilon _{v\,l,j-1}+\upvarepsilon _{v\,l,j-1}^p\right) \right) . \end{aligned}$$

This step is essentially inexpensive as the space functions are known and only a single time problem has to be solved for a given LATIN iteration *j*. However, just updating the time functions is usually not enough for the reduced order approximation. Therefore, sometimes it is necessary to expand the reduced order basis according to section 4.1. For a particular iteration *j*, after the time functions are updated, a criterion $\wp$ is calculated as

55 $$\begin{aligned} \wp _j&=\dfrac{\Vert e_j- e_{j-1}\Vert }{\dfrac{1}{2}\left( \Vert e_j \Vert +\Vert e_{j-1} \Vert \right) }, \text {with}\;\nonumber \\ e_j&=\left[ \dfrac{\int _0^T\int _\Omega \left( \left( \underline{\underline{\sigma }}_{v\,j}-\underline{\underline{\sigma }}_{u\,j}\right) :\left( \underline{\underline{\sigma }}_{v\,j}-\underline{\underline{\sigma }}_{u\,j}\right) \right) d\Omega dt}{\dfrac{1}{2}\int _0^T\int _\Omega \left( \underline{\underline{\sigma }}_{v\,j}:\underline{\underline{\sigma }}_{v\,j}+\underline{\underline{\sigma }}_{u\,j}:\underline{\underline{\sigma }}_{u\,j}\right) d\Omega dt}\right] ^{1/2}. \end{aligned}$$

If $\wp _j$ is less than a predetermined value, new space-time modes are added for the next iteration $j+1$.

For a given iteration *j*, the quantities are written as

56 $$\begin{aligned} \underline{\lambda }_{j}=\underline{\lambda }_{0}+\sum _{j^\prime =1}^{j}{\varDelta }\underline{\lambda }_{j^\prime }, \underline{u}_{j}=\underline{u}_{0}+\sum _{j^\prime =1}^{j}{\varDelta }\underline{u}_{j^\prime }. \end{aligned}$$

Denoting by $\varpi$ and $\varsigma$ the total numbers of modes generated till this iteration, then eq. (56) can be rewritten as

57 $$\begin{aligned} \underline{\lambda }_{j}=\underline{\lambda }_{0}+\sum _{\imath =1}^{\varpi }\underline{\overline{{\varLambda }}}^\imath \alpha ^\imath _\lambda , \underline{u}_{j}=\underline{u}_{0}+\sum _{\jmath =1}^{\varsigma }\underline{\overline{U}}^\jmath \alpha ^\jmath _u. \end{aligned}$$

Defining the cumulative difference operator as $\overline{\Delta }\bullet =\sum _{j^\prime =1}^{j}{\varDelta }\bullet _{j^\prime }$, eqs. (56) and (57) indicate that

58 $$\begin{aligned} \overline{\Delta }\underline{\lambda }=\sum _{\imath =1}^{\varpi }\underline{\overline{{\varLambda }}}^\imath \alpha ^\imath _\lambda , \overline{\Delta }\underline{u}=\sum _{\jmath =1}^{\varsigma }\underline{\overline{U}}^\jmath \alpha ^\jmath _u. \end{aligned}$$

The corresponding strains are written as

59 $$\begin{aligned} \underline{\underline{\varepsilon }}_{\lambda \,j}=\underline{\underline{\varepsilon }}_{\lambda \,0}+\overline{\Delta }\underline{\underline{\varepsilon }}_{\lambda } , \underline{\underline{\varepsilon }}_{u\,j}=\underline{\underline{\varepsilon }}_{u\,0}+\overline{\Delta }\underline{\underline{\varepsilon }}_{u}, \end{aligned}$$

with

60 $$\begin{aligned} \overline{\Delta }\underline{\underline{\varepsilon }}_{\lambda }=\underline{\underline{\varepsilon }}\left( \overline{\Delta }\underline{\lambda }\right) , \overline{\Delta }\underline{\underline{\varepsilon }}_{u}=\underline{\underline{\varepsilon }}\left( \overline{\Delta }\underline{u}\right) . \end{aligned}$$

The strain corresponding to $\underline{v}$ is obtained as

61 $$\begin{aligned} \underline{\underline{\varepsilon }}_{v\,j}=\underline{\underline{\varepsilon }}_{v\,0}+\overline{\Delta }\underline{\underline{\varepsilon }}_{v}, \end{aligned}$$

with

62 $$\begin{aligned} \overline{\Delta }\underline{\underline{\varepsilon }}_{v}=\overline{\Delta }\underline{\underline{\varepsilon }}_{v}^p-\overline{\Delta }\underline{\underline{\varepsilon }}_{u}^p+\overline{\Delta }\underline{\underline{\varepsilon }}_{u}+\overline{\Delta }\underline{\underline{\varepsilon }}_{\lambda }. \end{aligned}$$

The corresponding displacement $\underline{v}$ is calculated by solving the discretised equation

63 $$\begin{aligned} \left[ \mathbb {K}_{aa}\right] \left\{ \overline{\Delta }\textsf{v}_{a}\right\} {=}\left\{ \mathbb {K}_{aa} \overline{\Delta }\textsf{u}_{a}{+}\mathbb {K}_{aa}\overline{\Delta }\uplambda _{a}{+}\mathbb {L}_{ag}\left( \overline{\Delta }\upvarepsilon ^p_{v}{-}\overline{\Delta }\upvarepsilon ^p_{u}\right) \right\} , \end{aligned}$$

and thereafter using

64 $$\begin{aligned} \underline{v}_{j}=\underline{v}_{0}+\overline{\Delta }\underline{v}. \end{aligned}$$

For the local stage, no ROM is used and the process is as discussed in section 3.1.2, so is the stopping criterion of the LATIN iteration. It has also to be mentioned that for the initialisation part of the LATIN iteration, no PGD is used, and the methodology is as described in section 3.1. The PGD procedure has been described in algorithm 2.

**Algorithm 2:**
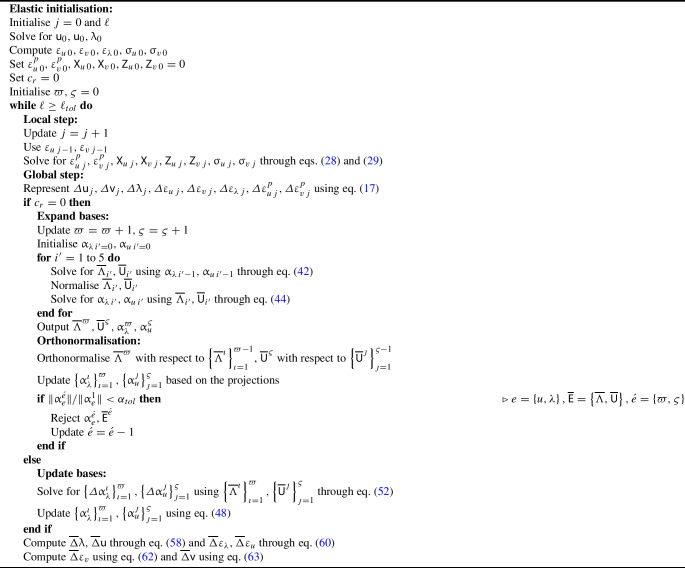
The basic problem procedure using PGD


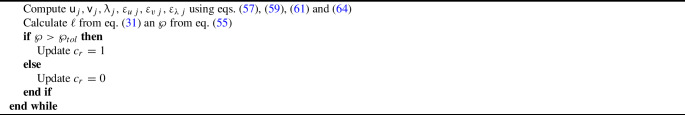


## Extension to softening behaviour

The previously described methodology is valid when the Hooke tensor $\boldsymbol{\textrm{C}}$ is constant. In case of damage, the elasticity (Young) modulus *E* is affected by the damage variable *D* as $\tilde{E}=E\left( 1-D\right)$ (where $\tilde{E}$ is the effective modulus), therefore the Hooke tensor is also affected. As the damage variable is a function of space and time, so is the Hooke tensor, therefore an alternative strategy for the problem formulation and resolution is necessary.

For the free energy function, consider the linear elastic case of eq. (16), which in the presence of damage becomes

65 $$\begin{aligned}  &   \psi \left( \underline{\underline{\varepsilon }}-\underline{\underline{\varepsilon }}^p, D, \underline{\underline{X}}\right) =\psi _\varepsilon \left( \underline{\underline{\varepsilon }}-\underline{\underline{\varepsilon }}^p, D\right) +\psi _X\left( \underline{\underline{X}}\right) , \text {with}\;\nonumber \\  &   \quad \psi _\varepsilon =\dfrac{1}{2}\left( \underline{\underline{\varepsilon }}-\underline{\underline{\varepsilon }}^p\right) :\boldsymbol{\textrm{C}}\left( 1-D\right) \left( \underline{\underline{\varepsilon }}-\underline{\underline{\varepsilon }}^p\right) . \end{aligned}$$

The associated or dual variable of *D* is obtained as

66 $$\begin{aligned} \breve{Y}=\dfrac{\partial \psi _\varepsilon }{\partial D}=-\dfrac{1}{2}\left( \underline{\underline{\varepsilon }}-\underline{\underline{\varepsilon }}^p\right) :\boldsymbol{\textrm{C}}\left( \underline{\underline{\varepsilon }}-\underline{\underline{\varepsilon }}^p\right) . \end{aligned}$$

The variable $-\breve{Y}$ is half of the variation in elastic energy generated by a damage variation at constant stress (see [28] for details), and is referred as strain energy release rate. For the sake convenience, a new variable *Y* is introduced such that $Y=-\breve{Y}$. This variable *Y*, i.e. the strain energy release rate, is considered to be the new dual (or thermodynamic force) of *D*, (see [29, 30] for details). It has also to be mentioned that *Y* can also be expressed in terms of stress, damage, triaxility function and triaxility ratio, as given in [30]. The corresponding evolution law is given as

67 $$\begin{aligned} \dot{D}=-\dfrac{\partial \phi ^*}{\partial \breve{Y}}, \text {or}\; \dot{D}=\dfrac{\partial \phi ^*}{\partial Y}. \end{aligned}$$

The variable set $\varkappa$ is then expanded to $\varkappa =\left\{ \underline{\underline{\varepsilon }}^e\left( =\underline{\underline{\varepsilon }}-\underline{\underline{\varepsilon }}^p\right) ,\right. \left. \underline{\underline{\varepsilon }}^p,\underline{\underline{\sigma }},\underline{\underline{X}},\underline{\underline{Z}}, D, Y\right\}$, and the residual errors defined in eq. (6) becomes

68a $$\begin{aligned}&\eta _\psi =\psi \left( \underline{\underline{\varepsilon }}^e,\underline{\underline{X}},D\right) + \psi ^*\left( \underline{\underline{\sigma }},\underline{\underline{Z}},Y\right) \nonumber \\  &\quad - \underline{\underline{\sigma }}:\underline{\underline{\varepsilon }}^e- \underline{\underline{Z}}\cdot \underline{\underline{X}}-YD, \end{aligned}$$

68b $$\begin{aligned}&\eta _\phi =\phi \left( \dot{\underline{\underline{\varepsilon }}}^p,-\dot{\underline{\underline{X}}},\dot{D}\right) + \phi ^*\left( \underline{\underline{\sigma }},\underline{\underline{Z}},Y\right) \nonumber \\  &\quad - \underline{\underline{\sigma }}:\dot{\underline{\underline{\varepsilon }}}^p+ \underline{\underline{Z}}\cdot \dot{\underline{\underline{X}}}-Y\dot{D}. \end{aligned}$$

### Classical formulation

To establish the methodology for the resolution of the basic problem, the attempt is first to develop the MCRE formulation without any model reduction. Following the descriptions of eq. section 3.1, the variables associated to $\underline{v}$ are $\varkappa _v=\left\{ \underline{\underline{\varepsilon }}_v^e\left( =\underline{\underline{\varepsilon }}_v-\underline{\underline{\varepsilon }}_v^p\right) ,\underline{\underline{\varepsilon }}_v^p,\underline{\underline{\sigma }}_v,\underline{\underline{X}}_v,\underline{\underline{Z}}_v, D_v, Y_v\right\}$, and variables associated to $\underline{u}$ are $\varkappa _u=\left\{ \underline{\underline{\varepsilon }}_u^e\left( =\underline{\underline{\varepsilon }}_u-\underline{\underline{\varepsilon }}_u^p\right) ,\underline{\underline{\varepsilon }}_u^p,\underline{\underline{\sigma }}_u,\underline{\underline{X}}_u,\right. \left. \underline{\underline{Z}}_u, D_u, Y_u\right\}$.

Equation (14) is rewritten as

69 $$\begin{aligned} \mathcal {L}&=\int _0^T\int _\Omega \left[ \psi \left( \underline{\underline{\varepsilon }}_u{-}\underline{\underline{\varepsilon }}_u^p,D_u,\underline{\underline{X}}_u\right) {-}\psi \left( \underline{\underline{\varepsilon }}_v-\underline{\underline{\varepsilon }}_v^p,D_v,\underline{\underline{X}}_v\right) \right. \nonumber \\&\quad \left. -\underline{\underline{\sigma }}_v:\left( \left( \underline{\underline{\varepsilon }}_u-\underline{\underline{\varepsilon }}_u^p\right) -\left( \underline{\underline{\varepsilon }}_v-\underline{\underline{\varepsilon }}_v^p\right) \right) \right. \nonumber \\&\left. -Y_v\left( D_u-D_v \right) -\underline{\underline{Z}}_v\cdot \left( \underline{\underline{X}}_u-\underline{\underline{X}}_v\right) \right] d\Omega dt \nonumber \\&\quad + \dfrac{\gamma }{2}\int _0^T\int _{\Omega _m} \left\| \underline{u}-\tilde{\underline{u}} \right\| ^2 d\Omega _m dt \nonumber \\&- \int _0^T\left[ \int _\Omega \underline{\underline{\sigma }}_v:\underline{\underline{\varepsilon }}\left( \underline{\lambda }\right) d\Omega - \int _\Omega \underline{f}_d^v\cdot \underline{\lambda }d\Omega \right. \nonumber \\&\quad \left. - \int _{\partial _f\Omega }\underline{f}_d^s\cdot \underline{\lambda }dS \right] dt. \end{aligned}$$

Similar to section 3.1.1, internal variables $\underline{\underline{X}}_u$, $\underline{\underline{X}}_v$, $\underline{\underline{Z}}_u$, $\underline{\underline{Z}}_v$, $\underline{\underline{\varepsilon }}_u^p$, $\underline{\underline{\varepsilon }}_v^p$, $D_u$, $D_v$, $Y_u$, $Y_v$ at the global step are frozen to the value obtained at the last local step.

From eq. (69), eq. (15) can be rewritten as

70a $$\begin{aligned}&\int _0^T\int _\Omega \boldsymbol{\textrm{C}}_v\left( \underline{\underline{\varepsilon }}_v-\underline{\underline{\varepsilon }}_v^p \right) :\underline{\underline{\varepsilon }}\left( \delta \underline{\lambda } \right) d\Omega dt \nonumber \\&\quad - \int _0^T\int _\Omega \underline{f}_d^v \cdot \delta \underline{\lambda } d\Omega dt - \int _0^T\int _{\partial _f\Omega }\underline{f}_d^s\cdot \delta \underline{\lambda } dS dt = 0, \end{aligned}$$

70b $$\begin{aligned}&\int _0^T\int _\Omega \boldsymbol{\textrm{C}}_v \left[ \left( \underline{\underline{\varepsilon }}_v-\underline{\underline{\varepsilon }}_v^p \right) -\left( \underline{\underline{\varepsilon }}_u-\underline{\underline{\varepsilon }}_u^p \right) -\underline{\underline{\varepsilon }}_{\lambda }\right] :\underline{\underline{\varepsilon }}\left( \delta \underline{v}\right) d\Omega dt=0, \end{aligned}$$

70c $$\begin{aligned}&\int _0^T\int _\Omega \left[ \boldsymbol{\textrm{C}}_u\left( \underline{\underline{\varepsilon }}_u-\underline{\underline{\varepsilon }}_u^p \right) -\boldsymbol{\textrm{C}}_v \left( \underline{\underline{\varepsilon }}_v-\underline{\underline{\varepsilon }}_v^p \right) \right] :\underline{\underline{\varepsilon }}\left( \delta \underline{u}\right) d\Omega dt \nonumber \\&\quad + \gamma \int _0^T\int _{\Omega _m} \left( \underline{u}-\tilde{\underline{u}} \right) \cdot \delta \underline{u}d\Omega _m dt=0, \end{aligned}$$

where

71 $$\begin{aligned} \boldsymbol{\textrm{C}}_v=\boldsymbol{\textrm{C}}\left( 1-D_v\right) , \boldsymbol{\textrm{C}}_u=\boldsymbol{\textrm{C}}\left( 1-D_u\right) . \end{aligned}$$

Following the definition of the corrective terms given by eq. (17) for a particular LATIN iteration *j*, eq. (70b) becomes

72 $$\begin{aligned}  &   \int _0^T\int _\Omega \boldsymbol{\textrm{C}}_{v\,j} \left[ \left( {\varDelta }\underline{\underline{\varepsilon }}_{v\,j}-{\varDelta }\underline{\underline{\varepsilon }}_{v\,j}^p \right) -\left( {\varDelta }\underline{\underline{\varepsilon }}_{u\,j}-{\varDelta }\underline{\underline{\varepsilon }}_{u\,j}^p \right) -{\varDelta }\underline{\underline{\varepsilon }}_{\lambda \,j}\right] \nonumber \\    &   \quad :\underline{\underline{\varepsilon }}\left( \delta \underline{v}\right) d\Omega dt=0, \end{aligned}$$

with

73 $$\begin{aligned} \boldsymbol{\textrm{C}}_{v\,j}=\boldsymbol{\textrm{C}}\left( 1-D_{v\,j}\right) . \end{aligned}$$

Moreover, eq. (21) can be rewritten as

74a $$\begin{aligned}&\int _0^T\int _\Omega \boldsymbol{\textrm{C}}_{v\,j}\left[ \left( \underline{\underline{\varepsilon }}_{v\,j-1}-\underline{\underline{\varepsilon }}_{v\,j-1}^p \right) \right. \nonumber \\  &\quad \left. +\left( {\varDelta }\underline{\underline{\varepsilon }}_{u\,j}-{\varDelta }\underline{\underline{\varepsilon }}_{u\,j}^p \right) +{\varDelta }\underline{\underline{\varepsilon }}_{\lambda \,j}\right] :\underline{\underline{\varepsilon }}\left( \delta \underline{\lambda } \right) d\Omega dt \nonumber \\&- \int _0^T\int _\Omega \underline{f}_d^v \cdot \delta \underline{\lambda } d\Omega dt - \int _0^T\int _{\partial _f\Omega }\underline{f}_d^s\cdot \delta \underline{\lambda } dS dt = 0, \end{aligned}$$

74b $$\begin{aligned}&\int _0^T\int _\Omega \left[ \boldsymbol{\textrm{C}}_{u\,j}\left( \underline{\underline{\varepsilon }}_{u\,j-1}-\underline{\underline{\varepsilon }}_{u\,j-1}^p \right) - \boldsymbol{\textrm{C}}_{v\,j}\left( \underline{\underline{\varepsilon }}_{v\,j-1}-\underline{\underline{\varepsilon }}_{v\,j-1}^p \right) \right. \nonumber \\  &\quad \left. +\left( \boldsymbol{\textrm{C}}_{u\,j}-\boldsymbol{\textrm{C}}_{v\,j}\right) \left( \Delta \underline{\underline{\varepsilon }}_{u\,j} \right. \right. \nonumber \\&\quad -\left. \left. \Delta \underline{\underline{\varepsilon }}_{u\,j}^p \right) - \boldsymbol{\textrm{C}}_{v\,j}{\varDelta }\underline{\underline{\varepsilon }}_{\lambda \,j} \right] :\underline{\underline{\varepsilon }}\left( \delta \underline{u}\right) d\Omega dt\nonumber \\  &\quad + \gamma \int _0^T\int _{\Omega _m} \left( \underline{u}_{j-1}+{\varDelta } \underline{u}_{j}-\tilde{\underline{u}} \right) \cdot \delta \underline{u}d\Omega _m dt=0. \end{aligned}$$

The discretised system (eq. (23)) becomes

75 $$\begin{aligned}  &   \begin{bmatrix} \mathbb {K}_{v\,aa,l,j} &  \mathbb {K}_{v\,aa,l,j}\\ \left( \gamma \Pi ^T\Pi \right) _{aa} +\mathbb {K}_{u\,aa,l,j} -\mathbb {K}_{v\,aa,l,j} &  - \mathbb {K}_{v\,aa,l,j} \end{bmatrix} \begin{bmatrix} {\varDelta }\textsf{u}_{a,l,j}\\ {\varDelta }\uplambda _{a,l,j} \end{bmatrix} \nonumber \\    &   \quad = \begin{bmatrix} \textsf{P}\\ \textsf{Q} \end{bmatrix}, \end{aligned}$$

with

76a $$\begin{aligned} \textsf{P}&=\mathbb {L}_{v\,ag,l,j}{\varDelta }\upvarepsilon _{u\,l,j}^p+\textsf{F}_a-\mathbb {L}_{v\,ag,l,j}\left( \upvarepsilon _{v\,l,j-1}-\upvarepsilon _{v\,l,j-1}^p\right) , \end{aligned}$$

76b $$\begin{aligned} \textsf{Q}&=\gamma \left( \left( \Pi ^T\right) _{ao}\tilde{\textsf{u}}_l- \left( \Pi ^T\Pi \right) _{ao} \textsf{u}_{l,j-1}\right) \nonumber \\  &\quad - \mathbb {L}_{u\,ag,l,j}\left( \upvarepsilon _{u\,l,j-1}-\upvarepsilon _{u\,l,j-1}^p-{\varDelta }\upvarepsilon _{u\,l,j}^p\right) \nonumber \\&\quad +\mathbb {L}_{v\,ag,l,j}\left( \upvarepsilon _{v\,l,j-1}-\upvarepsilon _{v\,l,j-1}^p-{\varDelta }\upvarepsilon _{u\,l,j}^p\right) , \end{aligned}$$

where the finite element operators are defined as

77 $$\begin{aligned}&\mathbb {K}_{v\,l,j}=\underset{\text {assembled}}{\left[ \int _\Omega \mathbb {B}^T \boldsymbol{\textrm{C}}\left( 1-\textsf{D}_{v\,l,j}\right) \mathbb {B} d\Omega \right] },\nonumber \\&\mathbb {K}_{u\,l,j}=\underset{\text {assembled}}{\left[ \int _\Omega \mathbb {B}^T \boldsymbol{\textrm{C}}\left( 1-\textsf{D}_{u\,l,j}\right) \mathbb {B} d\Omega \right] }, \nonumber \\&\mathbb {L}_{v\,l,j}=\underset{\text {assembled}}{\left[ \int _\Omega \mathbb {B}^T \boldsymbol{\textrm{C}}\left( 1-\textsf{D}_{v\,l,j}\right) d\Omega \right] },\nonumber \\&\mathbb {L}_{u\,l,j}=\underset{\text {assembled}}{\left[ \int _\Omega \mathbb {B}^T \boldsymbol{\textrm{C}}\left( 1-\textsf{D}_{u\,l,j}\right) d\Omega \right] }. \end{aligned}$$

The resolution of eq. (75) provides ${\varDelta }\textsf{u}_{j}, {\varDelta }\uplambda _{j}$, from which the corresponding strains ${\varDelta }\upvarepsilon _{v\,j}, {\varDelta }\upvarepsilon _{\lambda \,j}$ are obtained. Here $\textsf{D}$ is the discretised form of *D*.

The corrective term ${\varDelta }\textsf{v}_{j}$ is obtained by solving

78 $$\begin{aligned} \left[ \mathbb {K}_{v\,aa,l,j}\right] \left\{ {\varDelta }\textsf{v}_{a,l,j}\right\}= &   \left\{ \mathbb {K}_{v\,aa,l,j} {\varDelta }\textsf{u}_{a,l,j}\right. \nonumber \\    &   \quad \left. +\mathbb {K}_{v\,aa,l,j}{\varDelta }\uplambda _{a,l,j}\right. \nonumber \\  &   \left. +\mathbb {L}_{v\,ag,l,j}\left( {\varDelta }\upvarepsilon ^p_{v\,l,j}-{\varDelta }\upvarepsilon ^p_{u\,l,j}\right) \right\} , \end{aligned}$$

and the corresponding strain ${\varDelta }\upvarepsilon _{v\,j}$ is obtained from eq. (72).

It has to be mentioned that the initialisation step ($j=0$) is to be computed as given in section 3.1.1. Also, it has to be mentioned that the finite element operators (eq. (77)) are to be recomputed at each LATIN iteration *j* and at every time step, which exacerbates the computation cost of the problem resolution.

The local step is similar to section 3.1.2. For a particular time step $t_l$, at a particular LATIN iteration *j*, and for a particular Gauss point, with $w=\left\{ u,v \right\}$ the elastic prediction is given by

79a $$\begin{aligned}&\upvarepsilon ^p_{w\,l,j}=\upvarepsilon ^p_{w\,l-1,j}, \textsf{X}_{w\,l,j}=\textsf{X}_{w\,l-1,j}, \textsf{D}_{w\,l,j}=\textsf{D}_{w\,l-1,j}, \end{aligned}$$

79b $$\begin{aligned}&\textsf{Z}_{w\,l,j}=\dfrac{\partial \psi _X}{\partial \underline{\underline{X}}}\vert _{\textsf{X}_{w\,l,j}}, \upsigma _{w\,l,j}=\dfrac{\partial \psi _\varepsilon }{\partial \left( \underline{\underline{\varepsilon }}-\underline{\underline{\varepsilon }}^p\right) }\vert _{\upvarepsilon _{w\,l,j-1}, \upvarepsilon ^p_{w\,l,j}, \textsf{D}_{w\,l,j}}, \nonumber \\&\textsf{Y}_{w\,l,j}=-\dfrac{\partial \psi _\varepsilon }{\partial D}\vert _{\upvarepsilon _{w\,l,j-1}, \upvarepsilon ^p_{w\,l,j}}. \end{aligned}$$

The plastic correction is given as

80a $$\begin{aligned}&\upsigma _{w\,l,j}=\dfrac{\partial \psi _\varepsilon }{\partial \left( \underline{\underline{\varepsilon }}-\underline{\underline{\varepsilon }}^p\right) }\vert _{\upvarepsilon _{w\,l,j-1}, \upvarepsilon ^p_{w\,l,j}},\nonumber \\&\textsf{Z}_{w\,l,j}=\dfrac{\partial \psi _X}{\partial \underline{\underline{X}}}\vert _{\textsf{X}_{w\,l,j}}, \textsf{Y}_{w\,l,j}=-\dfrac{\partial \psi _\varepsilon }{\partial D}\vert _{\upvarepsilon _{w\,l,j-1}, \upvarepsilon ^p_{w\,l,j}} \end{aligned}$$

80b $$\begin{aligned}&\upvarepsilon ^p_{w\,l,j}=\upvarepsilon ^p_{w\,l-1,j} +{\varDelta } t_l \dfrac{\partial \phi ^*_p}{\partial \underline{\underline{\sigma }}}\vert _{\upsigma _{w\,l,j}, \textsf{Z}_{w\,l,j}, \textsf{D}_{w\,l,j}},\nonumber \\&\textsf{X}_{w\,l,j}=\textsf{X}_{w\,l-1,j} -{\varDelta } t_l \dfrac{\partial \phi ^*_p}{\partial \underline{\underline{Z}}}\vert _{\upsigma _{w\,l,j}, \textsf{Z}_{w\,l,j}, \textsf{D}_{w\,l,j}}, \nonumber \\&\textsf{D}_{w\,l,j}=\textsf{D}_{w\,l-1,j} +{\varDelta } t_l \dfrac{\partial \phi ^*_d}{\partial Y}\vert _{\textsf{Y}_{w\,l,j}}, \end{aligned}$$

where it is assumed that $\phi ^*\left( \underline{\underline{\sigma }}, \underline{\underline{Z}}, D, Y \right) =\phi ^*_p\left( \underline{\underline{\sigma }}, \underline{\underline{Z}}, D\right) +\phi ^*_d\left( Y\right)$, according to [31]; however other variations are also possible. Here $\textsf{Y}$ is the discretised form of *Y*.

For the initialisation step ($j=0$), the internal variables $\textsf{Y}_{u\,0},\textsf{Y}_{v\,0}$ are computed using eq. (66), while other internal variables $\upvarepsilon ^p_{u\,0},\upvarepsilon ^p_{v\,0},\textsf{X}_{u\,0},\textsf{X}_{v\,0},\textsf{Z}_{u\,0},\textsf{Z}_{v\,0}, \textsf{D}_{u\,0},\textsf{D}_{v\,0}$ are considered to be 0, and the stress variables are obtained from eq. (30).

The stopping criterion for the LATIN iterations is similar to section 3.1.2.

### ROM-based formulation

Similar to the formulations introduced in section 4, the intention herein is to propose a PGD based formulation with separated space and time problems for the global step. Consider the space and time problems given in section 4.1. Equation (38), can be rewritten for damageable behaviour as

81a $$\begin{aligned}&\int _\Omega \left[ \left( \int _0^T\boldsymbol{\textrm{C}}_{v\,j}\alpha _u \alpha _\lambda dt \right) \overline{\underline{\underline{\varepsilon }}}_u +\left( \int _0^T\boldsymbol{\textrm{C}}_{v\,j}\alpha _\lambda \alpha _\lambda dt\right) \overline{\underline{\underline{\varepsilon }}}_\lambda \right] \nonumber \\  &\quad :\underline{\underline{\varepsilon }}\left( \delta \underline{\overline{{\varLambda }}} \right) d\Omega \nonumber \\&\quad +\int _\Omega \left( \int _0^T\boldsymbol{\textrm{C}}_{v\,j}\left( \underline{\underline{\varepsilon }}_{v\,j-1}- \underline{\underline{\varepsilon }}_{v\,j-1}^p \right. \right. \nonumber \\&\quad \left. \left. -{\varDelta }\underline{\underline{\varepsilon }}_{u\,j}^p \right) \alpha _\lambda dt \right) :\underline{\underline{\varepsilon }}\left( \delta \underline{\overline{{\varLambda }}} \right) d\Omega \nonumber \\  &\quad - \int _\Omega \left( \int _0^T\underline{f}_d^v \alpha _\lambda dt \right) \cdot \delta \underline{\overline{{\varLambda }}} d\Omega \nonumber \\&\quad - \int _{\partial _f\Omega }\left( \int _0^T\underline{f}_d^s\alpha _\lambda dt\right) \cdot \delta \underline{\overline{{\varLambda }}} dS = 0, \end{aligned}$$

81b $$\begin{aligned}&\int _\Omega \left( \int _0^T \left( \boldsymbol{\textrm{C}}_{u\,j}- \boldsymbol{\textrm{C}}_{v\,j}\right) \alpha _u \alpha _u dt\right) \overline{\underline{\underline{\varepsilon }}}_u : \underline{\underline{\varepsilon }}\left( \delta \underline{\overline{U}}\right) d\Omega \nonumber \\  &\quad +\gamma \int _{\Omega _m} \left( \int _0^T \alpha _u \alpha _u dt \right) \underline{\overline{U}}\cdot \delta \underline{\overline{U}} d\Omega _m \nonumber \\&\quad - \int _\Omega \left( \int _0^T \boldsymbol{\textrm{C}} _v \alpha _u \alpha _\lambda dt\right) \overline{\underline{\underline{\varepsilon }}}_\lambda : \underline{\underline{\varepsilon }}\left( \delta \underline{\overline{U}}\right) d\Omega \nonumber \\  &\quad + \int _\Omega \left( \int _0^T\boldsymbol{\textrm{C}}_{u\,j}\left( \underline{\underline{\varepsilon }}_{u\,j-1}- \underline{\underline{\varepsilon }}_{u\,j-1}^p -{\varDelta } \underline{\underline{\varepsilon }}_{u\,j}^p \right) \alpha _u dt\right) \nonumber \\  &\quad : \underline{\underline{\varepsilon }}\left( \delta \underline{\overline{U}}\right) d\Omega \nonumber \\&\quad - \int _\Omega \left( \int _0^T\boldsymbol{\textrm{C}}_{v\,j}\left( \underline{\underline{\varepsilon }}_{v\,j-1}- \underline{\underline{\varepsilon }}_{v\,j-1}^p -{\varDelta } \underline{\underline{\varepsilon }}_{u\,j}^p \right) \alpha _u dt \right) \nonumber \\  &\quad : \underline{\underline{\varepsilon }}\left( \delta \underline{\overline{U}}\right) d\Omega \nonumber \\&\quad + \gamma \int _{\Omega _m} \left( \int _0^T\left( \underline{u}_{j-1}- \tilde{\underline{u}} \right) \alpha _u dt \right) \cdot \delta \underline{\overline{U}} d\Omega _m=0 . \end{aligned}$$

The corresponding discretised system becomes

82 $$\begin{aligned}  &   \begin{bmatrix} \mathbb {J}_{v\,aa,j} &  \mathbb {O}_{v\,aa,j}\\ \left( \gamma \Pi ^T\Pi \right) _{aa}\varrho +\mathbb {W}_{u\,aa,j} - \mathbb {W}_{v\,aa,j} &  - \mathbb {J}_{v\,aa,j} \end{bmatrix} \begin{bmatrix} \overline{\textsf{U}}_{a}\\ \overline{\Lambda }_{a} \end{bmatrix} \nonumber \\    &   \quad = \begin{bmatrix} \textsf{M}_a\\ \textsf{N}_a \end{bmatrix}, \end{aligned}$$

with

83a $$\begin{aligned} \mathbb {J}_{v,j}&=\lfloor \mathbb {K}_{v,l,j}\upalpha _{u\,l} \upalpha _{\lambda \,l} \rfloor , \mathbb {O}_{v,j}=\lfloor \mathbb {K}_{v,l,j}\upalpha _{\lambda \,l} \upalpha _{\lambda \,l} \rfloor , \end{aligned}$$

83b $$\begin{aligned} \mathbb {W}_{u,j}&=\lfloor \mathbb {K}_{u,l,j}\upalpha _{u\,l} \upalpha _{u\,l} \rfloor , \end{aligned}$$

83c $$\begin{aligned} \mathbb {W}_{v,j}&=\lfloor \mathbb {K}_{v,l,j}\upalpha _{u\,l} \upalpha _{u,l} \rfloor , \varrho =\lfloor \upalpha _{u\,l} \upalpha _{u\,l} \rfloor , \end{aligned}$$

83d $$\begin{aligned} \textsf{M}&=\lfloor \left( \mathbb {L}_{v,l,j}{\varDelta }\upvarepsilon _{u\,l,j}^p+\textsf{F}_l\right. \nonumber \\&\quad \left. - \mathbb {L}_{v,l,j}\left( \upvarepsilon _{v\,l,j-1}- \upvarepsilon _{v\,l,j-1}^p \right) \right) \upalpha _{\lambda \,l} \rfloor , \nonumber \\ \textsf{N}&=\lfloor \left( \gamma \left( \Pi ^T\tilde{\textsf{u}}_l- \Pi ^T\Pi \textsf{u}_{l,j-1}\right) \right. \nonumber \\&\quad \left. - \mathbb {L}_{u\,l,j}\left( \upvarepsilon _{u\,l,j-1}-\upvarepsilon _{u\,l,j-1}^p-{\varDelta }\upvarepsilon _{u\,l,j}^p\right) \right. \nonumber \\&\quad \left. +\mathbb {L}_{v\,l,j}\left( \upvarepsilon _{v\,l,j-1}-\upvarepsilon _{v\,l,j-1}^p-{\varDelta }\upvarepsilon _{u\,l,j}^p\right) \right) \upalpha _{u\,l} \rfloor . \end{aligned}$$

The time problem (eq. (40)) can also be rewritten as

84a $$\begin{aligned}&\int _0^T\left( \alpha _u \Upsilon +\alpha _\lambda \Gamma \right) \delta \alpha _\lambda dt \nonumber \\&\quad +\int _0^T\left( \int _\Omega \boldsymbol{\textrm{C}}_{v\,j}\left( \underline{\underline{\varepsilon }}_{v\,j-1}-\underline{\underline{\varepsilon }}_{v\,j-1}^p -{\varDelta }\underline{\underline{\varepsilon }}_{u\,j}^p \right) :\overline{\underline{\underline{\varepsilon }}}_\lambda d\Omega \right) \delta \alpha _\lambda dt \nonumber \\&\quad - \int _0^T \left( \int _\Omega \underline{f}_d^v\cdot \underline{\overline{{\varLambda }}} d\Omega \right) \delta \alpha _\lambda dt - \int _0^T \left( \int _{\partial _f\Omega }\underline{f}_d^s\cdot \underline{\overline{{\varLambda }}} dS\right) \delta \alpha _\lambda dt = 0, \end{aligned}$$

84b $$\begin{aligned}&\int _0^T \alpha _u \Psi \delta \alpha _u dt - \int _0^T \alpha _\lambda \Phi \delta \alpha _u dt \nonumber \\  &\quad + \int _0^T \left( \int _\Omega \boldsymbol{\textrm{C}}_{u\,j}\left( \underline{\underline{\varepsilon }}_{u\,j-1}-\underline{\underline{\varepsilon }}_{u\,j-1}^p -{\varDelta }\underline{\underline{\varepsilon }}_{u\,j}^p \right) : \overline{\underline{\underline{\varepsilon }}}_ud\Omega \right) \delta \alpha _u dt \nonumber \\  &\quad \quad - \int _0^T \left( \int _\Omega \boldsymbol{\textrm{C}}_{v\,j}\left( \underline{\underline{\varepsilon }}_{v\,j-1}-\underline{\underline{\varepsilon }}_{v\,j-1}^p -{\varDelta }\underline{\underline{\varepsilon }}_{u\,j}^p \right) : \overline{\underline{\underline{\varepsilon }}}_ud\Omega \right) \delta \alpha _u dt \nonumber \\&+ \gamma \int _0^T \left( \int _{\Omega _m} \left( \underline{u}_{j-1}-\tilde{\underline{u}} \right) \cdot \underline{\overline{U}} d\Omega _m\right) \delta \alpha _u dt =0 , \end{aligned}$$

with

85 $$\begin{aligned}&\Upsilon \left( t\right) =\int _\Omega \boldsymbol{\textrm{C}}_{v\,j}\overline{\underline{\underline{\varepsilon }}}_u : \overline{\underline{\underline{\varepsilon }}}_\lambda d\Omega , \Gamma \left( t\right) =\int _\Omega \boldsymbol{\textrm{C}}_{v\,j}\overline{\underline{\underline{\varepsilon }}}_\lambda : \overline{\underline{\underline{\varepsilon }}}_\lambda d\Omega , \nonumber \\&\Psi \left( t\right) =\int _\Omega \left( \boldsymbol{\textrm{C}}_{u\,j}-\boldsymbol{\textrm{C}}_{v\,j}\right) \overline{\underline{\underline{\varepsilon }}}_u : \overline{\underline{\underline{\varepsilon }}}_u d\Omega +\gamma \int _{\Omega _m}\underline{\overline{U}} \cdot \underline{\overline{U}} d\Omega _m, \nonumber \\&\Phi \left( t\right) =\int _\Omega \boldsymbol{\textrm{C}}_{v\,j}\overline{\underline{\underline{\varepsilon }}}_\lambda : \overline{\underline{\underline{\varepsilon }}}_u d\Omega . \end{aligned}$$

The discretised version (eq. (44)) then becomes

86 $$\begin{aligned} \begin{bmatrix} \Upsilon _l &  \Gamma _l\\ \Psi _l &  - \Phi _l \end{bmatrix} \begin{bmatrix} \upalpha _{u\,l}\\ \upalpha _{\lambda \,l} \end{bmatrix} = \begin{bmatrix} \textsf{G}_l\\ \textsf{H}_l \end{bmatrix}, \end{aligned}$$

with

87 $$\begin{aligned} \Upsilon _l= &   \overline{\Lambda }^T\mathbb {K}_{v\,l,j}\overline{\textsf{U}}, \Gamma _l=\overline{\Lambda }^T\mathbb {K}_{v\,l,j}\overline{\Lambda },\nonumber \\ \Psi _l= &   \overline{\textsf{U}}^T \left( \mathbb {K}_{u\,l,j}-\mathbb {K}_{v\,l,j}+\gamma \Pi ^T\Pi \right) \overline{\textsf{U}}, \Phi _l= \overline{\textsf{U}}^T\mathbb {K}_{v\,l,j}\overline{\Lambda }, \end{aligned}$$

and

88a $$\begin{aligned}&\textsf{G}_l=\overline{\Lambda }^T\left( \mathbb {L}_{v\,l,j}{\varDelta }\upvarepsilon _{u\,l,j}^p+\textsf{F}_l-\mathbb {L}_{v\,l,j}\left( \upvarepsilon _{v\,l,j-1}-\upvarepsilon _{v\,l,j-1}^p \right) \right) , \end{aligned}$$

88b $$\begin{aligned}&\textsf{H}_l= \overline{\textsf{U}}^T\left( \gamma \left( \Pi ^T\tilde{\textsf{u}}_l- \Pi ^T\Pi \textsf{u}_{l,j-1}\right) \right. \nonumber \\  &\quad \left. - \mathbb {L}_{u\,l,j}\left( \upvarepsilon _{u\,l,j-1}-\upvarepsilon ^p_{u\,l,j}-{\varDelta }\upvarepsilon _{u\,l,j}^p\right) \right. \nonumber \\&\quad \left. +\mathbb {L}_{v\,l,j}\left( \upvarepsilon _{v\,l,j-1}-\upvarepsilon ^p_{v\,l,j}-{\varDelta }\upvarepsilon _{u\,l,j}^p\right) \right) . \end{aligned}$$

The time problem for updating the reduced order bases (eq. (52)) becomes

89 $$\begin{aligned} \begin{bmatrix} \left[ \Upsilon \right] _l &  \left[ \Gamma \right] _l\\ \left[ \Psi \right] _l &  - \left[ \Phi \right] _l \end{bmatrix} \begin{bmatrix} \left\{ {\varDelta }\upalpha _u\right\} _l\\ \left\{ {\varDelta }\upalpha _\lambda \right\} _l \end{bmatrix} = \begin{bmatrix} \left\{ \textsf{G}\right\} _l\\ \left\{ \textsf{H}\right\} _l \end{bmatrix}, \end{aligned}$$

with

90 $$\begin{aligned}&\Upsilon _{\imath ,\jmath ,l}=\overline{\Lambda }^{\imath \,T}\mathbb {K}_{v\,l,j}\overline{\textsf{U}}^{\jmath }, \Gamma _{\imath ,\imath ^\prime ,l}=\overline{\Lambda }^{\imath \,T}\mathbb {K}_{v\,l,j}\overline{\Lambda }^{\imath ^\prime },\nonumber \\&\Psi _{\jmath ,\jmath ^\prime ,l}= \overline{\textsf{U}}^{\jmath \,T} \left( \mathbb {K}_{u\,l,j}-\mathbb {K}_{v\,l,j}+\gamma \Pi ^T\Pi \right) \overline{\textsf{U}}^{\jmath ^\prime }, \nonumber \\&\Phi _{\jmath ,\imath ,l}= \overline{\textsf{U}}^{\jmath \,T}\mathbb {K}_{v\,l,j}\overline{\Lambda }^{\imath }, \end{aligned}$$

and

91a $$\begin{aligned} \textsf{G}_{\imath ,l}&{=}\overline{\Lambda }^{\imath \,T}\left( \mathbb {L}_{v\,l,j}{\varDelta }\upvarepsilon _{u\,l,j}^p{+}\textsf{F}_l{-}\mathbb {L}_{v\,l,j}\left( \upvarepsilon _{v\,l,j{-}1}{-}\upvarepsilon _{v\,l,j-1}^p \right) \right) , \end{aligned}$$

91b $$\begin{aligned} \textsf{H}_{\jmath ,l}&= \overline{\textsf{U}}^{\jmath \,T}\left( \gamma \left( \Pi ^T\tilde{\textsf{u}}_l- \Pi ^T\Pi \textsf{u}_{l,j-1}\right) \right. \nonumber \\  &\quad \left. - \mathbb {L}_{u\,l,j}\left( \upvarepsilon _{u\,l,j-1}-\upvarepsilon ^p_{u\,l,j}-{\varDelta }\upvarepsilon _{u\,l,j}^p\right) \right. \nonumber \\&\quad \left. +\mathbb {L}_{v\,l,j}\left( \upvarepsilon _{v\,l,j-1}-\upvarepsilon ^p_{v\,l,j}-{\varDelta }\upvarepsilon _{u\,l,j}^p\right) \right) . \end{aligned}$$

The expansion of the reduced order bases, i.e. eqs. (82) and (86), can be resolved as per section 4.1. Similarly, eq. (89) can be solved as per section 4.2 to update the reduced order bases. However, the calculation of $\mathbb {J}_{v,j}, \mathbb {O}_{v,j}, \mathbb {W}_{u,j}, \mathbb {W}_{v,j}$ (eq. (83)) becomes expensive not only because of the integration, but also because $\mathbb {K}_{u,l,j}$ and $\mathbb {K}_{v,l,j}$ have to be computed at each $t_l$, where $l=\left\{ 0,\ldots ,f\right\}$. Also, for the time problem (eq. (86)), the matrix elements $\Upsilon _l, \Gamma _l, \Psi _l, \Phi _l$ are to be calculated at each time step $t_l$, which increases the computational cost further.

Consequently, it is necessary to reduce the fidelity of these matrices. To do so, after the local step as given in eqs. (79) and (80), singular value decompositions are performed on the snapshots $\textsf{I}-\textsf{D}_{u\,j}$ and $\textsf{I}-\textsf{D}_{v\,j}$, from which $\iota$ and $\mu$ modes are selected such that

92a $$\begin{aligned}&\textsf{I}-\textsf{D}_{u\,j}=\sum _{i^\prime =1}^\iota \overline{\textsf{D}}_{u}^{i^\prime } \upalpha _{d_u}^{i^\prime } \end{aligned}$$

92b $$\begin{aligned}&\textsf{I}-\textsf{D}_{v\,j}=\sum _{j^\prime =1}^\mu \overline{\textsf{D}}_{v}^{j^\prime } \upalpha _{d_v}^{j^\prime }, \end{aligned}$$

where $\overline{\textsf{D}}_{u}$ and $\overline{\textsf{D}}_{v}$ are the space functions, and $\upalpha _{d_u}$, $\upalpha _{d_v}$ are the time functions. It has to be mentioned that the number of modes to be selected is based on the magnitude of the singular values. The operators defined in eq. (77) can be rewritten in terms of spatial integrals for each mode as

93 $$\begin{aligned}&\overline{\mathbb {K}}_{v\,j^\prime ,j}=\underset{\text {assembled}}{\left[ \int _\Omega \mathbb {B}^T \boldsymbol{\textrm{C}}\left( 1-\overline{\textsf{D}}_{v}^{j^\prime }\right) \mathbb {B} d\Omega \right] },\nonumber \\&\overline{\mathbb {K}}_{u\,i^\prime ,j}=\underset{\text {assembled}}{\left[ \int _\Omega \mathbb {B}^T \boldsymbol{\textrm{C}}\left( 1-\overline{\textsf{D}}_{u}^{i^\prime }\right) \mathbb {B} d\Omega \right] }, \nonumber \\&\overline{\mathbb {L}}_{v\,j^\prime ,j}=\underset{\text {assembled}}{\left[ \int _\Omega \mathbb {B}^T \boldsymbol{\textrm{C}}\left( 1-\overline{\textsf{D}}_{v}^{j^\prime }\right) d\Omega \right] },\nonumber \\&\overline{\mathbb {L}}_{u\,i^\prime ,j}=\underset{\text {assembled}}{\left[ \int _\Omega \mathbb {B}^T \boldsymbol{\textrm{C}}\left( 1-\overline{\textsf{D}}_{u}^{i^\prime }\right) d\Omega \right] }, \end{aligned}$$

which define eq. (83) as

94a $$\begin{aligned} \mathbb {J}_{v,j}&=\sum _{j^\prime =1}^\mu \overline{\mathbb {K}}_{v,j^\prime ,j}\lfloor \upalpha _{d_v\,l}^{j^\prime } \upalpha _{u\,l} \upalpha _{\lambda \, l} \rfloor , \mathbb {O}_{v,j} \nonumber \\  &=\sum _{j^\prime =1}^\mu \overline{\mathbb {K}}_{v,j^\prime ,j}\lfloor \upalpha _{d_v\,l}^{j^\prime } \upalpha _{\lambda \,l} \upalpha _{\lambda \, l} \rfloor , \end{aligned}$$

94b $$\begin{aligned} \mathbb {W}_{u,j}&=\sum _{i^\prime =1}^\iota \overline{\mathbb {K}}_{u,i^\prime ,j}\lfloor \upalpha _{d_u\,l}^{i^\prime } \upalpha _{u\,l} \upalpha _{u \, l} \rfloor , \mathbb {W}_{v,j} \nonumber \\  &=\sum _{j^\prime =1}^\mu \overline{\mathbb {K}}_{v,j^\prime ,j}\lfloor \upalpha _{d_v\,l}^{j^\prime } \upalpha _{u\,l} \upalpha _{u \, l} \rfloor , \end{aligned}$$

94c $$\begin{aligned} \textsf{M}&=\sum _{j^\prime =1}^\mu \overline{\mathbb {L}}_{v,j^\prime ,j}\lfloor \left( {\varDelta }\upvarepsilon _{u\,l,j}^p-\upvarepsilon _{v\,l,j-1}+\upvarepsilon _{v\,l,j-1}^p \right) \upalpha _{d_v\,l}^{j^\prime }\nonumber \\  &\quad \upalpha _{\lambda \, l} \rfloor +\lfloor \textsf{F}_l \upalpha _{\lambda \, l} \rfloor , \end{aligned}$$

94d $$\begin{aligned} \textsf{N}&=\lfloor \gamma \left( \Pi ^T\tilde{\textsf{u}}_l- \Pi ^T\Pi \textsf{u}_{l,j-1}\right) \upalpha _{u \, l} \rfloor \nonumber \\&\quad - \sum _{i^\prime =1}^\iota \overline{\mathbb {L}}_{u\,i^\prime ,j}\lfloor \left( \upvarepsilon _{u\,l,j-1}-\upvarepsilon _{u\,l,j-1}^p-{\varDelta }\upvarepsilon _{u\,l,j}^p\right) \nonumber \\  &\quad \upalpha _{d_u\,l}^{i^\prime } \upalpha _{u \, l}\rfloor \nonumber \\&\quad +\sum _{j^\prime =1}^\mu \overline{\mathbb {L}}_{v\,j^\prime ,j}\lfloor \left( \upvarepsilon _{v\,l,j-1}-\upvarepsilon _{v\,l,j-1}^p-{\varDelta }\upvarepsilon _{u\,l,j}^p\right) \nonumber \\  &\quad \upalpha _{d_v\,l}^{j^\prime } \upalpha _{u \, l} \rfloor . \end{aligned}$$

As $\iota ,\mu \ll f$, the computation cost is reduced drastically.

Now,the quantities defined in eqs. (87) and (88) become

95 $$\begin{aligned}&\Upsilon _l=\sum _{j^\prime =1}^\mu \left( \overline{\Lambda }^T \overline{\mathbb {K}}_{v,j^\prime ,j}\overline{\textsf{U}}\right) \upalpha _{d_v\,l}^{j^\prime }, \nonumber \\  &\Gamma _l=\sum _{j^\prime =1}^\mu \left( \overline{\Lambda }^T \overline{\mathbb {K}}_{v,j^\prime ,j}\overline{\Lambda }\right) \upalpha _{d_v\,l}^{j^\prime },\nonumber \\&\Psi _l= \sum _{i^\prime =1}^\iota \left( \overline{\textsf{U}}^T \overline{\mathbb {K}}_{u\,i^\prime ,j}\overline{\textsf{U}}\right) \upalpha _{d_u\,l}^{i^\prime } \nonumber \\&-\sum _{j^\prime =1}^\mu \left( \overline{\textsf{U}}^T\overline{\mathbb {K}}_{v\,j^\prime ,j}\overline{\textsf{U}}\right) \upalpha _{d_v\,l}^{j^\prime }+\left( \gamma \overline{\textsf{U}}^T\Pi ^T\Pi \overline{\textsf{U}}\right) ,\nonumber \\&\Phi _l= \sum _{j^\prime =1}^\mu \left( \overline{\textsf{U}}^T\overline{\mathbb {K}}_{v\,j^\prime ,j}\overline{\Lambda }\right) \upalpha _{d_v\,l}^{j^\prime }, \end{aligned}$$

**Fig. 2:**
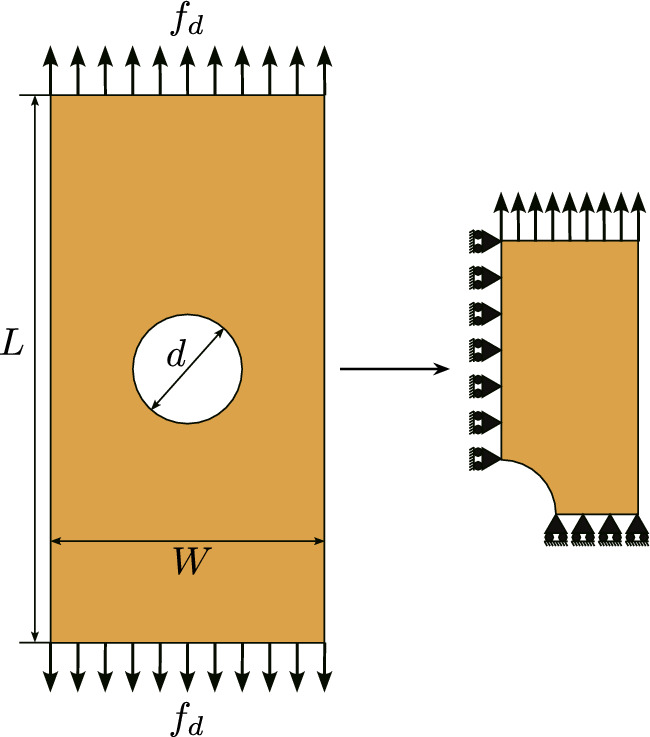
Rectangular plate with circular hole under traction

**Fig. 3:**
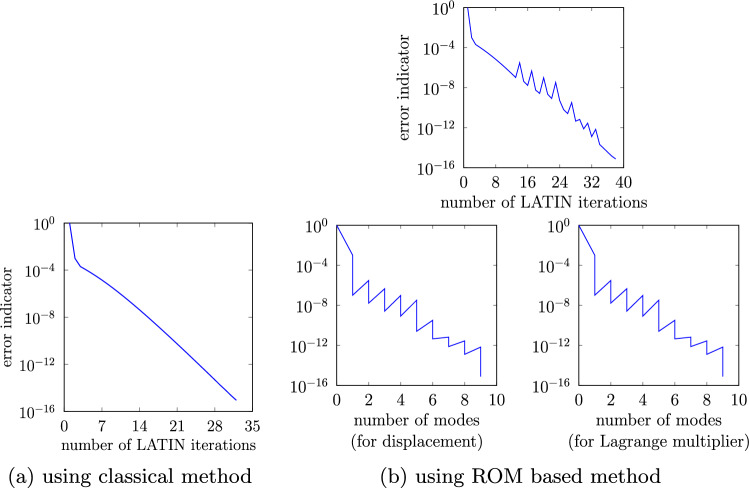
Error curves for the ${\textrm{MB}}_1$ solution

**Fig. 4:**
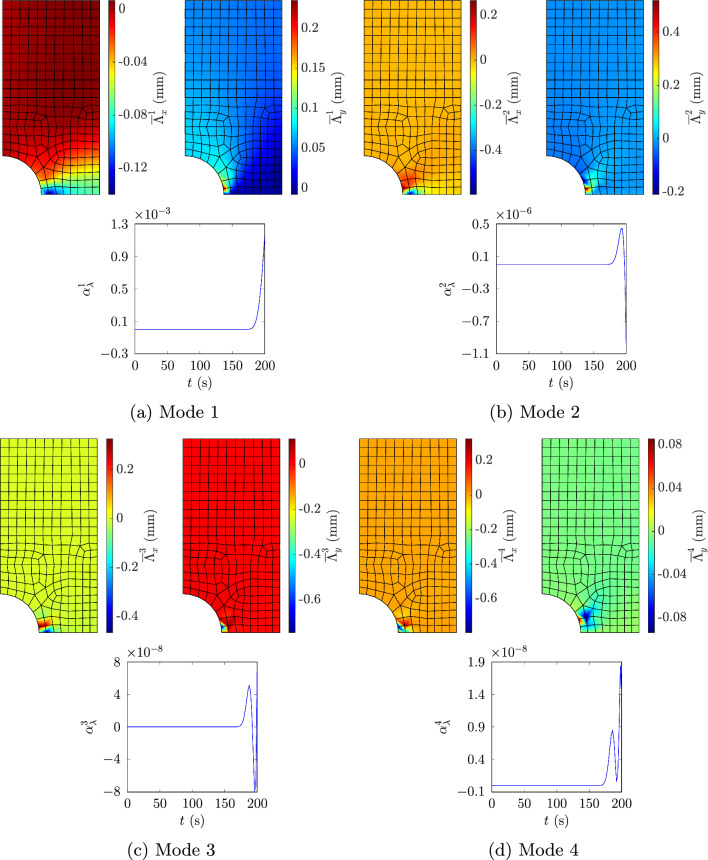
Initial space-time PGD modes describing the Lagrange multiplier $\underline{\lambda }$ for ${\textrm{MB}}_1$

**Fig. 5:**
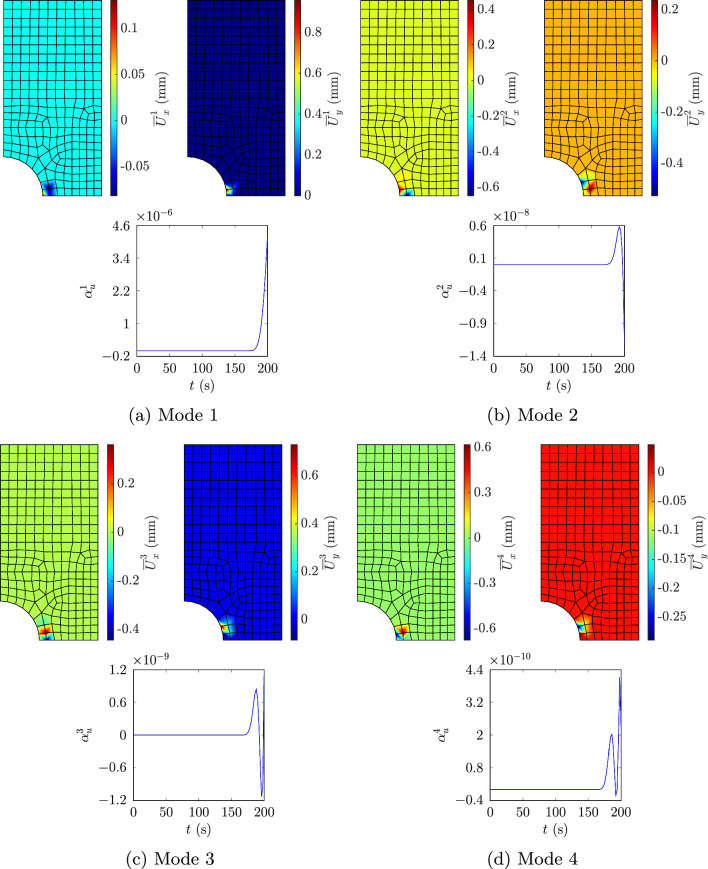
Initial space-time PGD modes describing the displacement $\underline{u}$ for ${\textrm{MB}}_1$

and

96a $$\begin{aligned}&\textsf{G}_l=\sum _{j^\prime =1}^\mu \overline{\Lambda }^T\overline{\mathbb {L}}_{v,j^\prime ,j}\left( {\varDelta }\upvarepsilon _{u\,l,j}^p-\upvarepsilon _{v\,l,j-1}+\upvarepsilon _{v\,l,j-1}^p \right) \upalpha _{d_v\,l}^{j^\prime } \nonumber \\  &\quad +\overline{\Lambda }^T \textsf{F}_l \end{aligned}$$

96b $$\begin{aligned}&\textsf{H}_l=\gamma \overline{\textsf{U}}^T\left( \Pi ^T\tilde{\textsf{u}}_l- \Pi ^T\Pi \textsf{u}_{l,j-1}\right) \nonumber \\&\quad - \sum _{i^\prime =1}^\iota \overline{\textsf{U}}^T\overline{\mathbb {L}}_{u\,i^\prime ,j}\left( \upvarepsilon _{u\,l,j-1}-\upvarepsilon _{u\,l,j-1}^p-{\varDelta }\upvarepsilon _{u\,l,j}^p\right) \upalpha _{d_u\,l}^{i^\prime } \nonumber \\&\quad +\sum _{j^\prime =1}^\mu \overline{\textsf{U}}^T\overline{\mathbb {L}}_{v\,j^\prime ,j}\left( \upvarepsilon _{v\,l,j-1}-\upvarepsilon _{v\,l,j-1}^p-{\varDelta }\upvarepsilon _{u\,l,j}^p\right) \upalpha _{d_v\,l}^{j^\prime } . \end{aligned}$$

It has to be mentioned that the solution cost of eqs. (95) and (96) is more than that of eqs. (87) and (88) (i.e. without SVD). However, as the solution of eq. (94) is much less than eq. (83), the overall process is cost effective compared to the resolution without SVD. To finish the development, eqs. (90) and (91) can be reformulated very easily similar to eqs. (95) and (96).

## Numerical results

For the numerical verification of the methodologies described before, a classical two-dimensional plate with a hole problem is considered (see fig. 2). The geometry of the plate is defined by the width $W= 100\,\textrm{mm}$, the length $L=200\,\textrm{mm}$, and the hole diameter $d=40\,\textrm{mm}$. A uniformly distributed, linearly increasing traction $f_d=150\left( t/T\right) \, \mathrm {N/mm}$ is applied on top and bottom surfaces, where $t\in \left[ 0,T\right]$ with $T=200\,\textrm{s}$.

Due to symmetry, one-fourth of the plate is considered for the study with symmetric boundaries as shown in fig. 2. Now, two types of material behaviour (MB) are considered for the following studies; one is the classical elasto-viscoplasticity with isotropic hardening (${\textrm{MB}}_1$), and the other is the elasto-viscoplasticity coupled with isotropic hardening and isotropic damage (${\textrm{MB}}_2$).

- ${\textrm{MB}}_1$- For elasto-viscoplasticity coupled with isotropic hardening, the free energy function is given as
  97 $$\begin{aligned} \psi \left( \underline{\underline{\varepsilon }}-\underline{\underline{\varepsilon }}^p, r\right) =\psi _\varepsilon \left( \underline{\underline{\varepsilon }}-\underline{\underline{\varepsilon }}^p\right) +\psi _r\left( r\right) , \end{aligned}$$
  with
  98 $$\begin{aligned} \psi _\varepsilon =\dfrac{1}{2}\left( \underline{\underline{\varepsilon }}-\underline{\underline{\varepsilon }}^p\right) :\boldsymbol{\textrm{C}}\left( \underline{\underline{\varepsilon }}-\underline{\underline{\varepsilon }}^p\right) , \psi _r=\dfrac{1}{2}r R_\infty r. \end{aligned}$$
  These provide the state equations
  99 $$\begin{aligned}&\underline{\underline{\sigma }}=\boldsymbol{\textrm{C}}\left( \underline{\underline{\varepsilon }}-\underline{\underline{\varepsilon }}^p\right) , R= R_\infty r. \end{aligned}$$
  Here $R_\infty$ is the hardening parameter that relates the drag strain (primal variable) *r* to the drag stress (dual variable) *R*. The Hooke tensor $\boldsymbol{\textrm{C}}\left( E,\nu \right)$ is described by the Young modulus *E* and Poisson’s ratio $\nu$. The pseudo dissipation potential in this case is given as per Norton’s law as
  100 $$\begin{aligned} \phi ^*=\dfrac{1}{n+1}\left\langle \dfrac{f_y}{K} \right\rangle ^{n+1}, \end{aligned}$$
  where the yield function given by von Mises criterion can be written as
  101 $$\begin{aligned} f_y=\left| \underline{\underline{\sigma }}^D\right| _{eq}-R-\sigma _y, \end{aligned}$$
  with $\vert \bigcirc \vert _{eq}=\sqrt{3/2\bigcirc :\bigcirc }$, $\sigma _y$ being the yield stress defining the elastic limit, and $\underline{\underline{\sigma }}^D$ being the deviatoric part of the stress tensor. *K* and *n* are the viscous coefficient and exponent, respectively. The evolution laws then become
  102 $$\begin{aligned}&\dot{\underline{\underline{\varepsilon }}}^p=K^{-n}\left\langle f_y \right\rangle ^{n}\left( \dfrac{3}{2} \dfrac{\underline{\underline{\sigma }}^D}{\vert \underline{\underline{\sigma }}^D\vert _{eq}} \right) , \dot{r}= K^{-n}\left\langle f_y \right\rangle ^{n}. \end{aligned}$$
  It has to mentioned here that the cumulative plastic strain *p*, defined as
  103 $$\begin{aligned} \dot{p}=\sqrt{\dfrac{2}{3}\dot{\underline{\underline{\varepsilon }}}^p:\dot{\underline{\underline{\varepsilon }}}^p}, \end{aligned}$$
  is basically the same as the drag strain, i.e
  104 $$\begin{aligned} \dot{p}=\dot{r}. \end{aligned}$$
- ${\textrm{MB}}_2$- For elasto-viscoplasticity coupled with isotropic hardening and isotropic damage, the free energy function is given as
  105 $$\begin{aligned} \psi \left( \underline{\underline{\varepsilon }}-\underline{\underline{\varepsilon }}^p, D,r\right) =\psi _\varepsilon \left( \underline{\underline{\varepsilon }}-\underline{\underline{\varepsilon }}^p,D\right) +\psi _r\left( r\right) , \end{aligned}$$
  with
  106 $$\begin{aligned} \psi _\varepsilon =\dfrac{1}{2}\left( \underline{\underline{\varepsilon }}-\underline{\underline{\varepsilon }}^p\right) :\boldsymbol{\textrm{C}}\left( 1-D\right) \left( \underline{\underline{\varepsilon }}-\underline{\underline{\varepsilon }}^p\right) , \psi _r=\dfrac{1}{2}r R_\infty r. \end{aligned}$$
  These provide the state equations
  107 $$\begin{aligned}&\underline{\underline{\sigma }}=\boldsymbol{\textrm{C}}\left( 1-D\right) \left( \underline{\underline{\varepsilon }}-\underline{\underline{\varepsilon }}^p\right) , \nonumber \\&Y=\dfrac{1}{2}\left( \underline{\underline{\varepsilon }}-\underline{\underline{\varepsilon }}^p\right) :\boldsymbol{\textrm{C}}\left( \underline{\underline{\varepsilon }}-\underline{\underline{\varepsilon }}^p\right) , \nonumber \\&R= R_\infty r. \end{aligned}$$

The dual potential is separated as (according to [32])

108 $$\begin{aligned} \psi ^*\left( \underline{\underline{\sigma }},R,D,Y\right) =\psi _p^*\left( \underline{\underline{\sigma }},R,D \right) +\phi _D^*\left( Y \right) , \end{aligned}$$

with

109 $$\begin{aligned}  &   \psi _p^*\left( \underline{\underline{\sigma }},R,D \right) =\dfrac{1}{n+1}\left\langle \dfrac{f_y}{K} \right\rangle ^{n+1}, \nonumber \\  &   \phi _D^*\left( Y \right) =\dfrac{1}{s+1}\left\langle \dfrac{Y-Y_{th}}{S} \right\rangle ^{s+1}, \end{aligned}$$

where $Y_{th}$ is the threshold value beyond which damage evolution occurs, and *s*, *S* are the material parameters dictating the damage law. The yield function for this case is given by

**Fig. 6:**
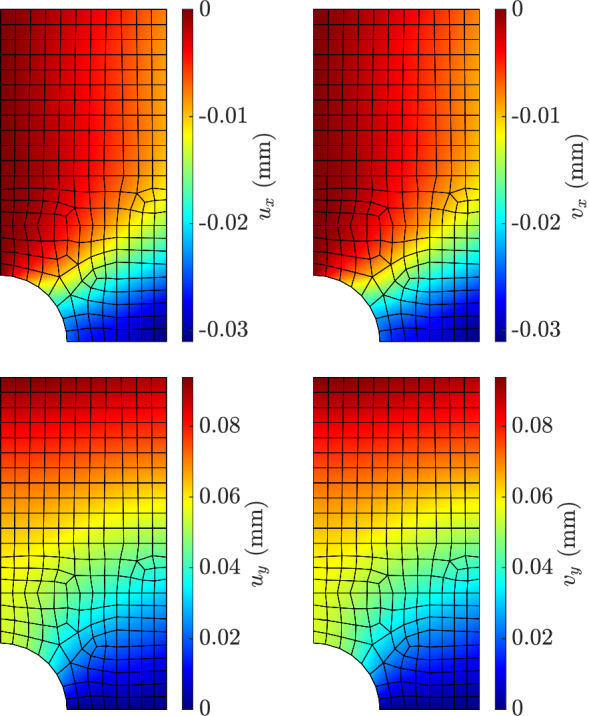
Displacement fields ($\underline{u},\underline{v}$) at the final time step for ${\textrm{MB}}_1$

**Fig. 7:**
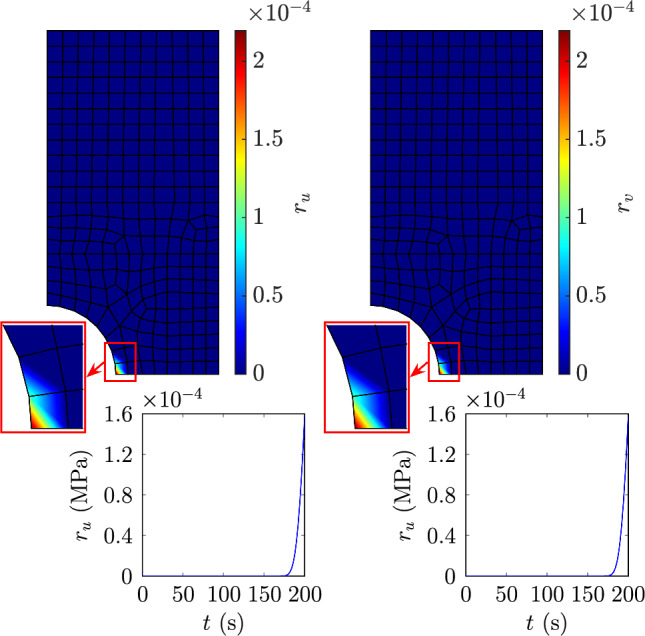
Fields of drag strains ($r_u, r_v$) at the final time step, and maximum drag strain evolutions for ${\textrm{MB}}_1$

110 $$\begin{aligned} f_y=\left| \dfrac{\underline{\underline{\sigma }}^D}{1-D}\right| _{eq}-R-\sigma _y. \end{aligned}$$

The evolution laws are given by

111 $$\begin{aligned}&\dot{\underline{\underline{\varepsilon }}}^p=K^{-n}\left\langle f_y \right\rangle ^{n}\left( \dfrac{3}{2} \dfrac{\dfrac{\underline{\underline{\sigma }}^D}{1-D}}{\left| \dfrac{\underline{\underline{\sigma }}^D}{1-D}\right| _{eq}} \right) \dfrac{1}{1-D} , \nonumber \\&\dot{r}= K^{-n}\left\langle f_y \right\rangle ^{n}, \dot{D}= S^{-s}\left\langle Y-Y_{th} \right\rangle ^{s}, \end{aligned}$$

and in the presence of damage

112 $$\begin{aligned} \dot{p}=\dfrac{\dot{r}}{1-D}. \end{aligned}$$

252 four-node quadrilateral elements, with four Gauss points per element, are used to discretise the structure to be studied. This generates a total of 288 nodes, with two degrees of freedom per node, and 1008 Gauss points. For the time discretisation, 101 time steps are used to discretise the time frame $\left[ 0,200\,\textrm{s}\right]$.

**Fig. 8:**
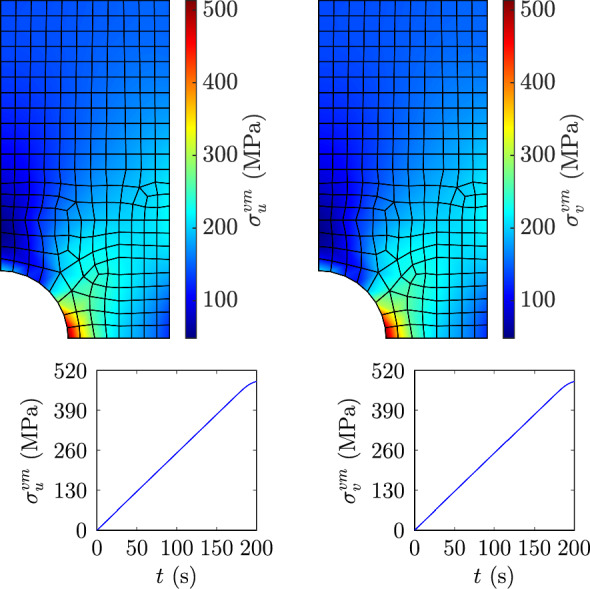
Fields of von Misses stresses ($\sigma _u^{vm}, \sigma _v^{vm}$) at the final time step, and maximum von Misses stress evolutions for ${\textrm{MB}}_1$

**Fig. 9:**
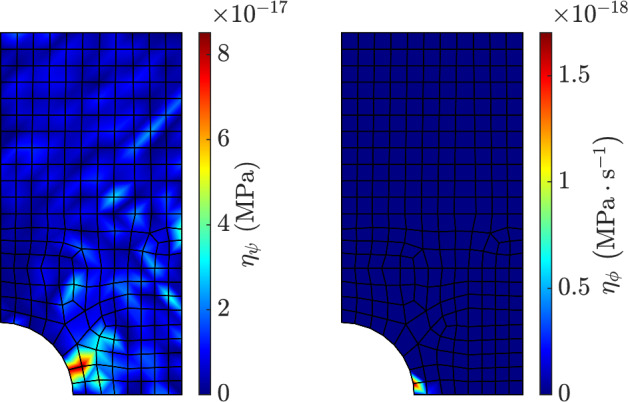
Fields of residual errors ($\eta _\psi , \eta _\phi$) at the final time step for ${\textrm{MB}}_1$

At first, direct problems are solved for both material behaviours in order to generate the synthetic data $\tilde{\textsf{u}}$. The reference material parameters used for the direct problem solutions are $E^{ref}=200\,\textrm{GPa}, \nu ^{ref}=0.3, \sigma _y^{ref}=400\,\textrm{MPa}, R_\infty ^{ref}=10\,\textrm{GPa},K^{ref}=400\,\textrm{MPa} \textrm{s}^{1/n}, n^{ref}=7, s^{ref}=2.5, S^{ref}=1.5\,\textrm{MPa} \textrm{s}^{1/s}, Y^{ref}_{th}=0.45\,\textrm{MPa}$.

Thereafter, for the inverse analyses, the intend is to perform two separate studies. The first one is to solve the basic problem using eq. (4) for ${\textrm{MB}}_1$ and using eq. (5) for ${\textrm{MB}}_2$. The second study is to perform complete inverse simulation using the total MCRE process including the ROMs.

**Fig. 10:**
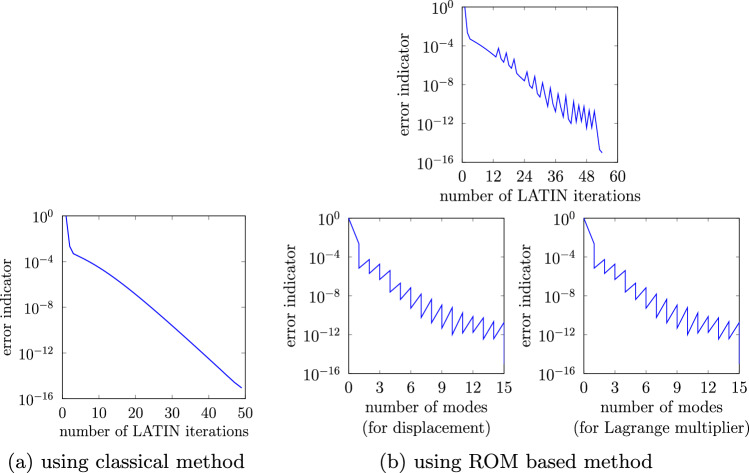
Error curves for the ${\textrm{MB}}_2$ solution

**Fig. 11:**
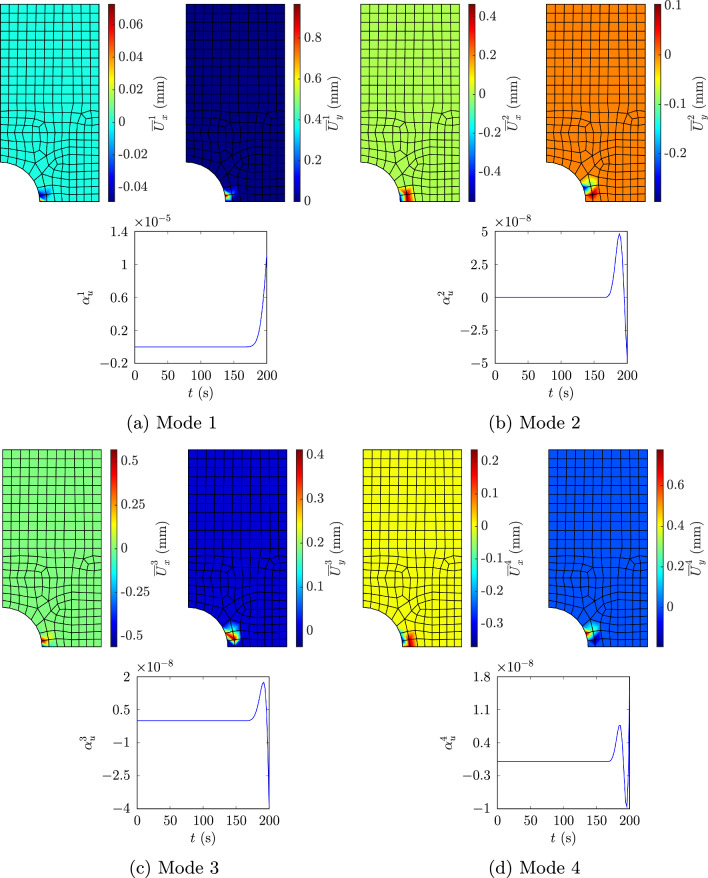
Initial space-time PGD modes describing the displacement $\underline{u}$ for ${\textrm{MB}}_2$

### Solving the basic problem

The discretisation for this part of the study is the same as that of the direct problem. The scaling factor $\gamma$ is considered to be $10^7$, based on Morozov’s principle (see [17] for details). Reference basic problem solutions (without any reduced order modelling) are performed using the reference material parameters, first for ${\textrm{MB}}_1$ through the process described in eq. section 3.1, and then for ${\textrm{MB}}_2$ through the procedure described in section 5.1. Thereafter ROM based formulations are used to solve the basic problem, according to section 4 for ${\textrm{MB}}_1$, and according to section 5.2 for ${\textrm{MB}}_2$.

For ${\textrm{MB}}_1$ ROM-based solution, the error curves are shown in fig. 3. They basically represent the LATIN error indicator (defined in eq. (31)) with respect to the LATIN iterations. Section 6.1 also depicts the evolution of the error indicator with respect to the number of PGD modes generated. A total of 9 PGD modes were generated to approximate both the displacement field and the Lagrange multiplier. As per the CPU time is concerned, the ROM-based solution time was observed to be $60\%$ of the solution time of the reference problem.

The initial four PGD modes used to approximate $\underline{\lambda }$ are shown in fig. 4, and the same for $\underline{u}$ is depicted in fig. 5.

The final displacement field for ${\textrm{MB}}_1$ is depicted in fig. 6, and the displacement fields $\underline{u}$ and $\underline{v}$ have errors of $3.5\times 10^{-15}\%$ and $2.9\times 10^{-13}\%$ with respect to the reference solutions. The error $er_{\bigcirc }$ of any quantity $\bigcirc$ is given as

113 $$\begin{aligned} er_{\bigcirc }=\left[ \dfrac{\int _0^T\int _\Omega \left( \left( \bigcirc -\bigcirc _{ref}\right) :\left( \bigcirc -\bigcirc _{ref}\right) \right) d\Omega dt}{\int _0^T\int _\Omega \left( \bigcirc _{ref}:\bigcirc _{ref}\right) d\Omega dt}\right] ^{1/2}. \end{aligned}$$

The drag strains $r_u, r_v$ at the final time step, for ${\textrm{MB}}_1$, are shown in fig. 7. This figure also shows the evolution of the maximum drag strains with respect to time. The errors for $r_u, r_v$ are obtained to be $3.1\times 10^{-11}\%, 5.5\times 10^{-11}\%$, and their corresponding duals $R_u, R_v$ provide the same error values.

The von Misses stresses $\sigma _u^{vm}, \sigma _v^{vm}$ at the final time step for ${\textrm{MB}}_1$, along with the time evolutions of their maximum values, are depicted in fig. 8. The stresses $\underline{\underline{\sigma }}_u, \underline{\underline{\sigma }}_v$ have errors of $3.2\times 10^{-13}\%$ and $3.7\times 10^{-13}\%$.

The errors corresponding to the plastic strains $\underline{\underline{\varepsilon }}^p_u, \underline{\underline{\varepsilon }}^p_v$ for ${\textrm{MB}}_1$ are obtained to be $3.1\times 10^{-11}\%, 5.3\times 10^{-11}\%$, and $3.4\times 10^{-13}\%, 4.9\times 10^{-13}\%$ for the total strains $\underline{\underline{\varepsilon }}_u, \underline{\underline{\varepsilon }}_v$.

The residual errors, defined in eq. (6) is plotted at the end of loading in fig. 9, and as expected the magnitudes are quiet low (as the basic problem is solved using reference parameters), and maximum values are concentrated at the plastified zone.

Similarly, the same exercise is conducted for ${\textrm{MB}}_2$. The error curves, for both classical solution and ROM-based solution are shown in fig. 10. 15 PGD modes were generated to approximate both $\underline{u}$ and $\underline{\lambda }$. The CPU time for the ROM-based solution was found to be $70\%$ of the classical resolution.

**Fig. 12:**
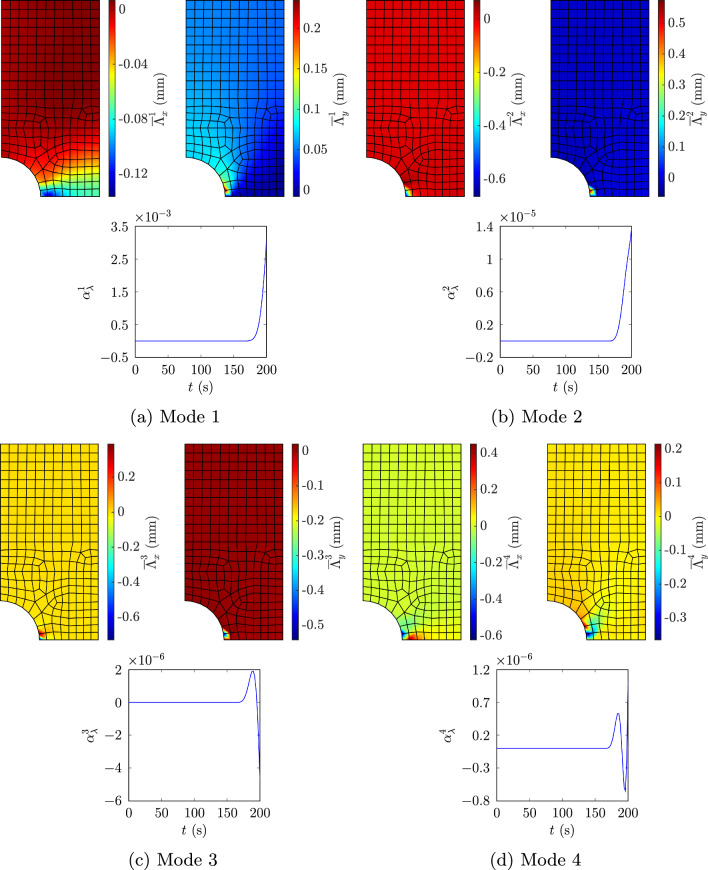
Initial space-time PGD modes describing the Lagrange multiplier $\underline{\lambda }$ for ${\textrm{MB}}_2$

The separated PGD modes (first four) for both $\underline{u}$ and $\underline{\lambda }$ are depicted in figs. 11 and 12.

**Fig. 13:**
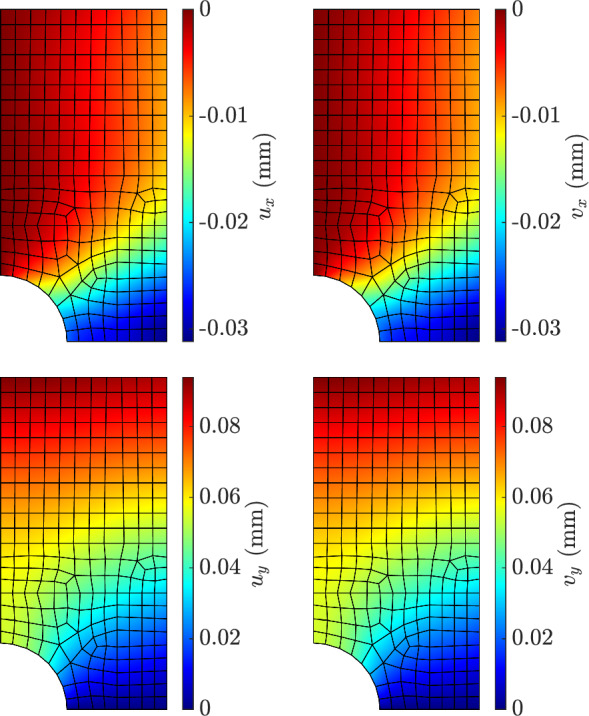
Displacement fields ($\underline{u},\underline{v}$) at the final time step for ${\textrm{MB}}_2$

The displacement fields $\underline{u},\underline{v}$ for ${\textrm{MB}}_2$ have depicted errors of $6.3\times 10^{-13}\%$ and $1.2\times 10^{-11}\%$, and the final field is shown in fig. 13.

**Fig. 14:**
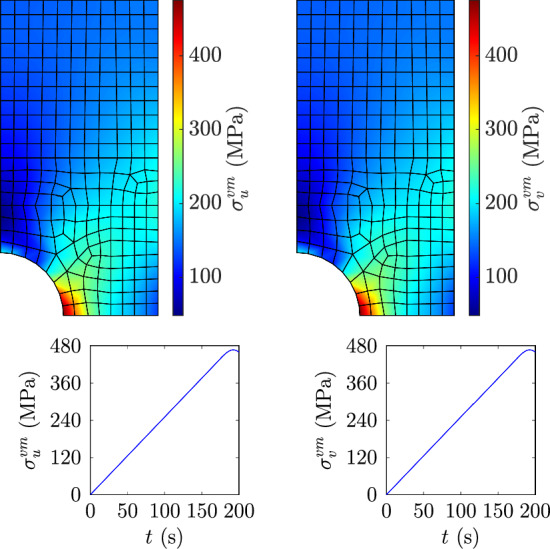
Fields of von Misses stresses ($\sigma _u^{vm}, \sigma _v^{vm}$) at the final time step, and maximum von Misses stress evolutions for ${\textrm{MB}}_2$

The von Misses stresses $\sigma _u^{vm}, \sigma _v^{vm}$ at the final time step for ${\textrm{MB}}_2$, along with the time evolutions of their maximum values are depicted in fig. 14. The stresses $\underline{\underline{\sigma }}_u, \underline{\underline{\sigma }}_v$ have errors of $1.5\times 10^{-11}\%, 5.5\times 10^{-10}\%$.

**Fig. 15:**
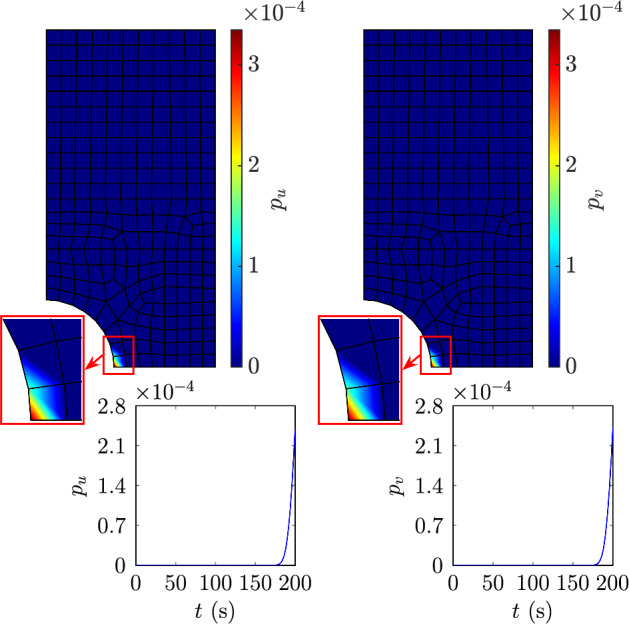
Fields of cumulative plastic strains ($p_u, p_v$) at the final time step, and maximum cumulative plastic strain evolutions for ${\textrm{MB}}_2$

The cumulative plastic strains $p_u, p_v$ at the final time step, for ${\textrm{MB}}_2$, are shown in fig. 15. This figure also shows the evolution of the maximum drag strains with respect to time. The errors corresponding to the plastic strains $\underline{\underline{\varepsilon }}^p_u, \underline{\underline{\varepsilon }}^p_v$ for ${\textrm{MB}}_2$ are obtained to be $2.9\times 10^{-11}\%, 4.9\times 10^{-11}\%$, and $1.6\times 10^{-11}\%, 6.9\times 10^{-10}\%$ for the total strains $\underline{\underline{\varepsilon }}_u, \underline{\underline{\varepsilon }}_v$.

**Fig. 16:**
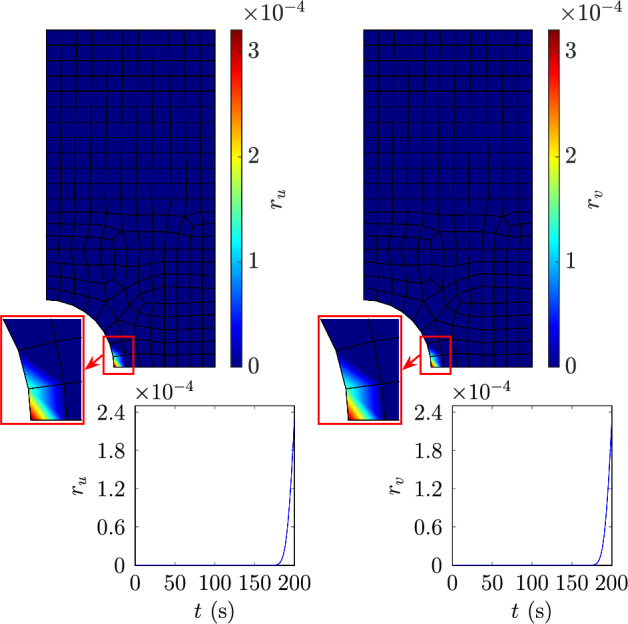
Fields of drag strains ($r_u, r_v$) at the final time step, and maximum drag strain evolutions for ${\textrm{MB}}_2$

The errors for $r_u, r_v$ are obtained to be $2.8\times 10^{-11}\%, 4.8\times 10^{-11}\%$ for ${\textrm{MB}}_2$, and their corresponding duals $R_u, R_v$ provide the same error values. $r_u, r_v$ at the final time step for ${\textrm{MB}}_2$, along with the time evolutions of their maximum values, are depicted in fig. 16.

**Fig. 17:**
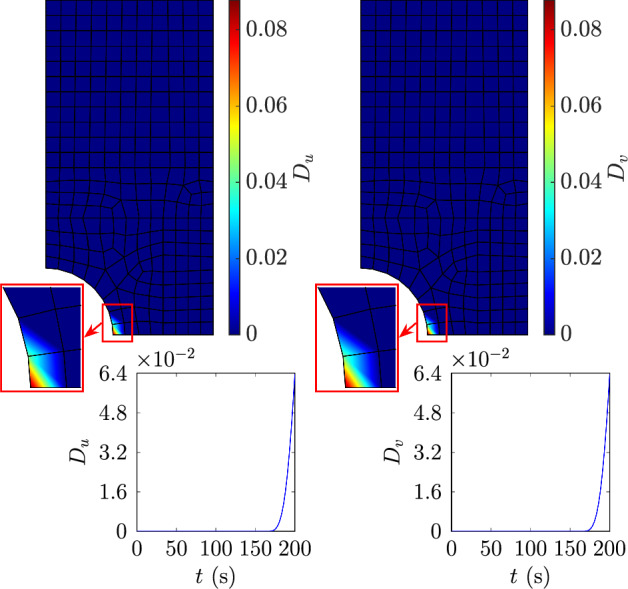
Fields of damage variables ($D_u, D_v$) at the final time step, and maximum damage evolutions for ${\textrm{MB}}_2$

The damage variables $D_u, D_v$ at the final time step for ${\textrm{MB}}_2$, along with the time evolutions of their maximum values, are depicted in fig. 17. Errors corresponding to $D_u, D_v$ are $1.3\times 10^{-11}\%, 1.7\times 10^{-11}\%$, and errors for $Y_u, Y_v$ are $2.6\times 10^{-11}\%, 8.3\times 10^{-10}\%$. It has to be mentioned that the 8 SVD modes were generated (for both $D_u, D_v$) from the snapshots of the damage variables (as given in section 5.2).

**Fig. 18:**
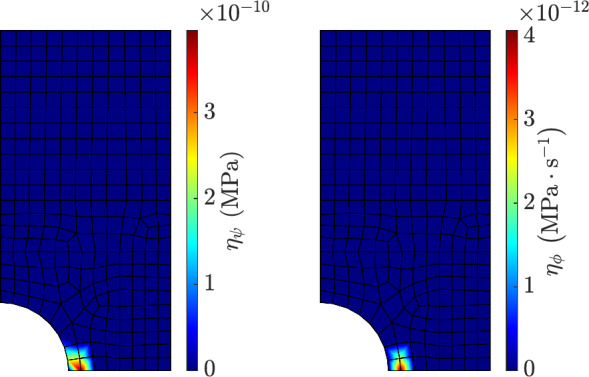
Fields of residual errors ($\eta _\psi , \eta _\phi$) at the final time step for ${\textrm{MB}}_2$

The residual errors, defined in eq. (68) is plotted at the end of loading in fig. 18, and similar to fig. 9 the magnitudes are quiet low, and maximum values are concentrated at the plastified (and damaged) zone.

From these analyses, it is seen that that admissible states provide excellent accuracy for the ROM-based solutions of the basic problem, compared to the classical solutions. Also the ROM-based solutions were found to be more frugal compared to the classical solutions.

### Identification of material parameters

In this part, the first exercise is to solve the total inverse problem, with the basic problem being solved through section 4 for ${\textrm{MB}}_1$, and through section 5 for ${\textrm{MB}}_2$. The elastic parameters ($E,\nu$) for both cases are considered to be known parameters.

**Fig. 19:**
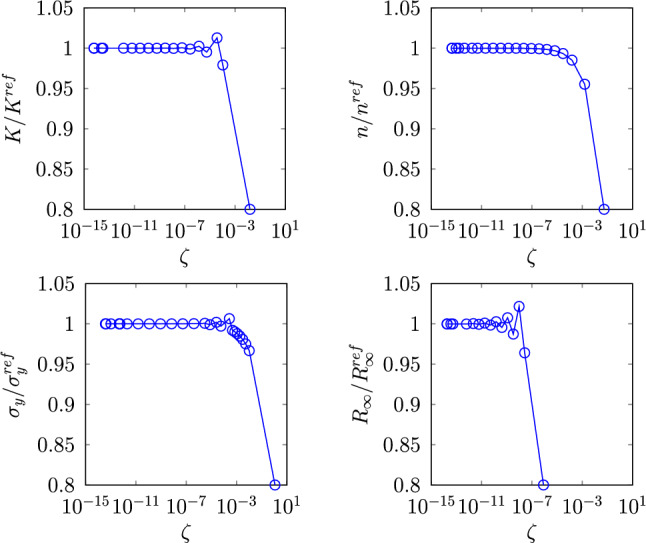
Convergence curves of material parameters with respect to the MCRE for ${\textrm{MB}}_1$

Figure 19 depicts the evolution of parameters for ${\textrm{MB}}_1$ at different gradient descent iterations, along with the corresponding MCRE functional values. It is clear from fig. 19 that the parameters converge to their reference values with successive iterations of the gradient descent algorithm, and the MCRE functionals decrease to their minimal values. Each of the material parameters shown in fig. 19 are identified individually, with the rest considered to be known and equal to reference values.

**Fig. 20:**
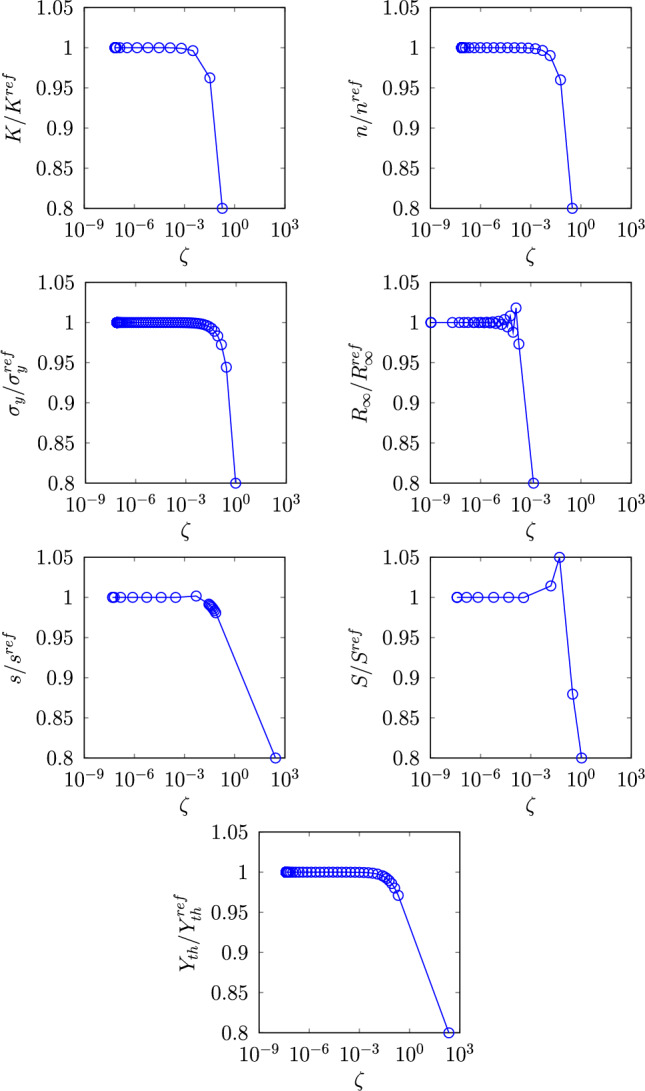
Convergence curves of material parameters with respect to the MCRE for ${\textrm{MB}}_2$

A similar exercise is performed for ${\textrm{MB}}_2$, as depicted in fig. 20. Similar to the previous case, each of the material parameters shown in fig. 20 are identified individually, with the rest considered to be known and equal to reference values.

**Fig. 21:**
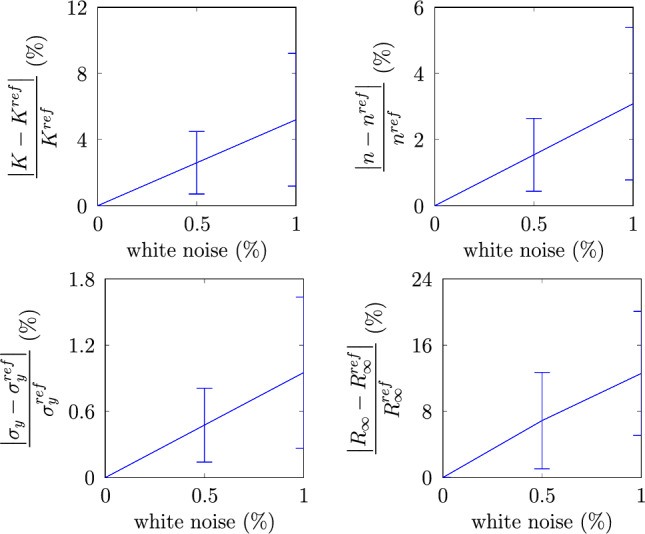
Accuracy and precision of identified material parameters with respect to the reference parameters for ${\textrm{MB}}_1$

**Fig. 22:**
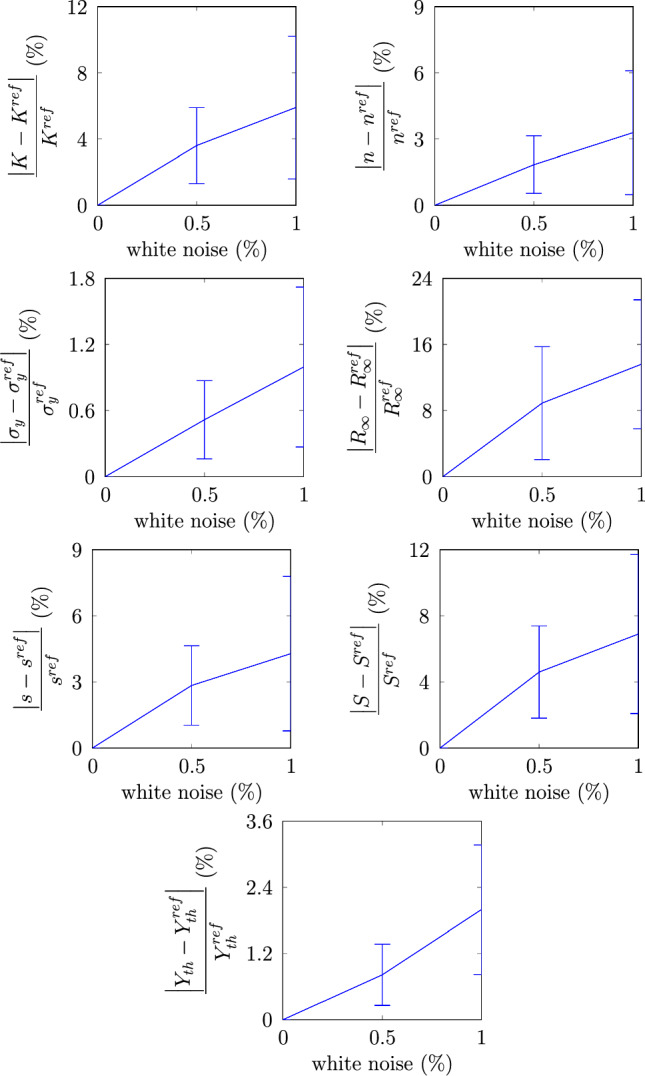
Accuracy and precision of identified material parameters with respect to the reference parameters for ${\textrm{MB}}_2$

Now, for the next set of numerical tests, percentage white noises are added as a spurious input in the measurement data as

114 $$\begin{aligned} \tilde{\underline{u}}=\tilde{\underline{u}}_0+\delta \tilde{\underline{u}}, \end{aligned}$$

where $\tilde{\underline{u}}_0$ is the measurement data from the direct problem and $\delta \tilde{\underline{u}}$ is a sample of Gaussian white noise. Different levels of Gaussian white noises ($0.5\%$ and $1\%$) are added to the non-spurious synthetic data from the direct problem resolutions. For each noise level, 10 different random samples (i.e. $\delta \tilde{\underline{u}}$) are used, and then the ROM based MCRE inverse analysis is performed to determine the parameters using the spurious data. The corresponding means and standard deviations at each noise level, of the errors with respect to the reference parameters are calculated (see figs. 21 and 22).

It is clear from fig. 21, that the accuracies and the precisions decrease with increase in noise level, also from fig. 21, it can be seen that $\sigma _y$ is most precisely and accurately identified, and $R_\infty$ is most susceptible to noise.

Similar to ${\textrm{MB}}_1$, it is clear from fig. 22, that the accuracies and the precisions decrease with increase in noise level. Also from fig. 22, it can be seen that $\sigma _y$ and $Y_{th}$ are the best identified parameters, and $R_\infty$ is the worst identified parameter.

These exercises were conducted considering the complete time domain, to establish the proof of concept of the ROM based basic problems in the overall framework of the inverse analysis.

## Conclusion

This article essentially depicted the accuracy and frugality of PGD reduced order models in the solution of MCRE-based inverse approaches for nonlinear model updating. The methodology has been tested on viscoplastic and coupled damage-viscoplastic material identifications, and the veracity along with numerical expense has been observed to be appreciable. For damageable behaviours, a POD-based model reduction method has also been coupled, to treat the variable Hooke tensor efficiently. The overall attempt to introduce ROM in MCRE has been quite successful. A further work will consist in implementing the proposed strategy in a sequential model updating method, using a modified dual Kalman filter introduced in [21, 33] and which leans on MCRE. In addition, an application of the proposed strategy with real data, as well as an extension to the dynamics/vibration regime, are forecast in the framework of an ongoing project dealing with real-time SHM [1].

## Data Availability

Data, materials, and code that support the findings of this study are available from the authors upon reasonable request.
